# MicroRNA Nobel Prize: Timely Recognition and High Anticipation of Future Products—A Prospective Analysis

**DOI:** 10.3390/ijms252312883

**Published:** 2024-11-29

**Authors:** Sarfaraz K. Niazi, Matthias Magoola

**Affiliations:** 1 College of Pharmacy, University of Illinois, Chicago, IL 60612, USA; 2 DEI Biopharma, Kampala P.O. Box 35854, Uganda; dei@deigroupinternational.com

**Keywords:** microRNA, RNA, Nobel Prize, mRNA inhibition, therapies, ex vivo manipulation, in vivo administration, regulatory process, intellectual property, off-target effects, development, testing

## Abstract

MicroRNAs (miRNAs) maintain cellular homeostasis by blocking mRNAs by binding with them to fine-tune the expression of genes across numerous biological pathways. The 2024 Nobel Prize in Medicine and Physiology for discovering miRNAs was long overdue. We anticipate a deluge of research work involving miRNAs to repeat the history of prizes awarded for research on other RNAs. Although miRNA therapies are included for several complex diseases, the realization that miRNAs regulate genes and their roles in addressing therapies for hundreds of diseases are expected; but with advancement in drug discovery tools, we anticipate even faster entry of new drugs. To promote this, we provide details of the current science, logic, intellectual property, formulations, and regulatory process with anticipation that many more researchers will introduce novel therapies based on the discussion and advice provided in this paper.

## 1. Introduction

MiRNAs are small, noncoding RNAs containing 21–23 nucleotides [1,2] and are found in plants, animals, and even some viruses. Since their discovery in the early 1990s, miRNAs have transformed our understanding of cellular processes, particularly how genes are regulated in various biological contexts, such as development, differentiation, cell proliferation, and disease. These short RNA molecules function by binding to complementary sequences on target messenger RNA (mRNA) transcripts, leading to mRNA degradation or the repression of translation. This mechanism allows for miRNAs to fine-tune the expression of genes across numerous biological pathways and ensure the proper functioning of cellular activities.

miRNAs are involved in RNA silencing and the posttranscriptional regulation of gene expression [3,4] by base-pairing to complementary sequences in mRNA molecules [4], modulating the translation or stability of these transcripts. MiRNA functions through its seed region, a short sequence (2–8 nucleotides) that binds to the complementary region in the target mRNA, typically within the 3′ untranslated region (3′ UTR). Once bound, miRNAs can either cause the degradation of the mRNA or inhibit its translation to proteins, depending on the degree of complementarity between the miRNA and the target mRNA. A near-perfect match typically leads to mRNA degradation, while partial complementarity represses translation [5].

MiRNAs are critical regulators in nearly all biological processes, including embryogenesis, cell cycle control, apoptosis, and immune responses. These tiny RNA molecules are often called “fine-tuners” of gene expression because they dampen the expression of specific target genes without entirely shutting down their function. This fine-tuning is essential for maintaining cellular homeostasis and responding to changing environmental conditions. For instance, miRNAs can rapidly adjust gene expression in response to stress or injury, making them crucial players in tissue repair and regeneration [6].

The discovery of miRNAs expanded the scope of gene regulation significantly beyond what was previously known from protein-coding genes. Before their identification, gene regulation was thought to be primarily controlled at the transcriptional level, where transcription factors either activated or repressed gene transcription from DNA to mRNA. However, identifying miRNAs revealed that gene expression could also be controlled posttranscriptionally, adding a new layer of regulation that allows for greater flexibility and control over cellular processes. This discovery was revolutionary, as it provided a new perspective on how cells manage the complexity of their gene regulatory networks [7].

The widespread impact of miRNAs on gene expression is illustrated by their evolutionary conservation across species. MiRNAs are found in almost all multicellular organisms, from plants to humans, suggesting that their regulatory roles are fundamental to life. For example, miR-1, one of the first miRNAs identified in mammals, plays a critical role in regulating muscle development and is conserved across many species, including fruit flies, worms, and humans. This conservation highlights the importance of miRNAs in controlling essential cellular functions and maintaining evolutionary stability [8].

In addition to their roles in normal biological processes, miRNAs have been implicated in various diseases. Dysregulation of miRNA expression is commonly associated with cancer, cardiovascular diseases, neurodegenerative disorders, and metabolic syndromes. For instance, miR-21 is often overexpressed in several types of cancers, including breast, lung, and colon cancers, where it acts as an oncogene by promoting cell proliferation and inhibiting apoptosis. Similarly, the downregulation of miR-34, a tumor-suppressor miRNA, is observed in many cancers, where its loss leads to uncontrolled cell growth and tumor formation. The involvement of miRNAs in such a wide range of diseases has made them attractive targets for therapeutic interventions. Researchers are now exploring restoring normal miRNA function or inhibiting harmful miRNAs to treat diseases [9]. 

## 2. MiRNA Nobel Prize

**Figure 1:**
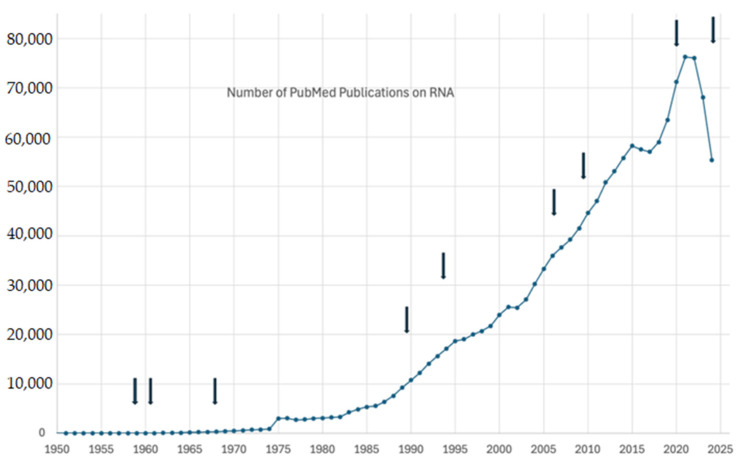
Publications on the topic of RNA; arrows point out the Nobel Prizes awarded in their fields. Arrows indicate Nobel Prizes awarded. (https://pubmed.ncbi.nlm.nih.gov/?term=RNA&sort=date) (accessed on 12 October 2024).

Nobel prizes bring instant recognition and add awareness of technologies, as we anticipate the 2024 award will do to the field of microRNAs [10]. To describe the state-of-the-art research on miRNAs, we are summarizing their properties as well as projecting their future applications. Interest in RNAs is well demonstrated by the number of publications listed in PubMed on this topic. The field of RNA has been very productive in bringing many remarkable contributions to molecular biology. Table 1 shows the various types of RNAs, their prevalence and recognition, where applicable, and other RNA inventions that have received Nobel Prizes (Figure 1).

The awarding of the Nobel Prize in Physiology or Medicine in 2024 to Victor Ambros and Gary Ruvkun for their pioneering work in the discovery and characterization of microRNAs (miRNAs) signifies the culmination of decades of research into small, noncoding RNA molecules that regulate gene expression. The journey to the Nobel Prize began in 1993, when Victor Ambros and his team at Harvard University, along with Gary Ruvkun at Massachusetts General Hospital, discovered lin-4, the first known miRNA, in the nematode *Caenorhabditis elegans*. This was one of the first insights into how miRNAs function to repress gene expression posttranscriptionally [6]. This discovery challenged the prevailing dogma that proteins exclusively controlled gene expression and opened the door to an entirely new realm of genetic regulation. Lin-4 was found to regulate the timing of *C. elegans’* development by repressing the expression of the lin-14 gene through a mechanism that did not involve protein-coding functions. Instead, lin-4 acted as a small RNA bound to complementary sequences in the 3′ untranslated region (3′ UTR) of lin-14 mRNA, thereby inhibiting its translation to protein. This was the first clear evidence that small, noncoding RNAs could regulate gene expression posttranscriptionally [8]. This discovery demonstrated, for the first time, that small, noncoding RNAs could control gene expression at the posttranscriptional level, a revolutionary concept at the time [5,9].

Although the discovery of lin-4 was groundbreaking, it was initially seen as an anomaly—something unique to nematodes. However, in year 2000, Ruvkun’s group made another seminal discovery: they identified let-7, a second miRNA, in *C. elegans*, which also regulated developmental timing. What made let-7 particularly significant was that it was evolutionarily conserved across species, from worms to humans. This finding demonstrated that miRNAs were not merely an oddity of nematode biology but a universal mechanism of gene regulation. The discovery of let-7 catalyzed a wave of research into miRNAs, as scientists realized that these small RNAs played critical roles in gene regulation across various organisms [11,12]. This finding broadened the scope of miRNA research and suggested that these small RNAs were not an evolutionary oddity but a fundamental part of gene regulation in complex organisms.

Thomas Tuschl and colleagues developed the first cloning and sequencing method to systematically identify miRNAs, discovering numerous new miRNAs in *Drosophila*, mice, and humans [13]. This methodological breakthrough allowed researchers to catalog miRNAs across different species and tissues, revealing that miRNAs are involved in virtually all cellular processes, including cell division, differentiation, apoptosis, and metabolism.

## 3. Nomenclature

Under a standard nomenclature system, names are assigned to experimentally confirmed miRNAs before publication [14,15]. The prefix “miR” is followed by a dash and a number, the latter often indicating the order of the naming. For example, miR-124 was named and likely discovered prior to miR-456. A capitalized “miR-” refers to the mature form of the miRNA, while the uncapitalized “mir-” refers to the pre-miRNA and the pri-miRNA [16]. The genes encoding miRNAs are also named using the same three-letter prefix, according to the conventions of the organism’s gene nomenclature. For example, some organisms’ official miRNA gene names are “*mir-1*” in *C. elegans* and *Drosophila*, “*Mir1*” in *Rattus norvegicus*, and *Mir25* in humans.

MiRNAs with nearly identical sequences except for one or two nucleotides are annotated with an additional lowercase letter. For example, miR-124a is closely related to miR-124b and

hsa-miR-181a: aacauucaACgcugucggugAgu and

hsa-miR-181b: aacauucaUUgcugucggugGgu.

Pre-miRNAs, pri-miRNAs, and genes leading to 100% identical mature miRNAs located at different places in the genome are indicated with an additional dash–number suffix. For example, the pre-miRNAs hsa-mir-194-1 and hsa-mir-194-2 lead to an identical mature miRNA (hsa-miR-194) but are from genes located in different genome regions.

Species of origin are designated with a three-letter prefix, e.g., hsa-miR-124 is a human (*Homo sapiens*) miRNA, and oar-miR-124 is a sheep (*Ovis aries*) miRNA. Other common prefixes include “v” for viral (miRNA encoded by a viral genome) and “d” for *Drosophila* miRNA (a fruit fly commonly studied in genetic research).

When two mature microRNAs originate from opposite arms of the same pre-miRNA and are found in roughly similar amounts, they are denoted with a -3p or -5p suffix. (In the past, this distinction was also made with “s” (sense) and “as” (antisense)). However, the mature microRNA found from one arm of the hairpin is usually much more abundant than that found from the other arm [3], in which case, an asterisk following the name indicates the mature species found at low levels from the opposite arm of a hairpin. For example, miR-124 and miR-124* share a pre-miRNA hairpin, but much more miR-124 is found in the cell. Table 2 lists the miRNA databases that list thousands of these identified miRNAs. 

## 4. Biogenesis

**Figure 2:**
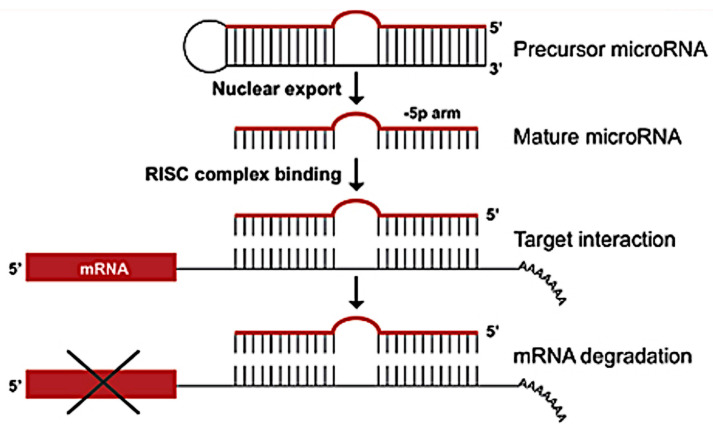
The general process of miRNA biogenesis.

Biogenesis, the synthesis of miRNA by living organisms, involves several distinct steps: transcription, nuclear processing, export, and cytoplasmic maturation (Figure 2).

The process of miRNA biogenesis begins with the transcription of miRNA genes by RNA polymerase II to long primary transcripts known as pri-miRNAs [17] (Figure 1). These pri-miRNAs are capped and polyadenylated, resembling typical mRNA transcripts. The pri-miRNA is then processed by a microprocessor complex consisting of Drosha, an RNase III enzyme, and its cofactor DGCR8 (DiGeorge syndrome critical region 8), which cleaves the pri-miRNA into a shorter precursor miRNA (pre-miRNA), approximately 70 nucleotides long. This pre-miRNA has a characteristic hairpin structure that is essential for its recognition and further processing. Once the pre-miRNA is produced, it is exported from the nucleus to the cytoplasm by Exportin-5, a transporter protein that recognizes the hairpin structure [6].

In the cytoplasm, the pre-miRNA is further processed by the Dicer enzyme, another RNase III protein, which cleaves the hairpin loop of the pre-miRNA to generate a double-stranded RNA molecule approximately 20–25 nucleotides in length. This duplex consists of two strands: the guide strand of mature miRNA and the passenger strand (or miRNA*), which is typically degraded. The guide strand is then incorporated into the RNA-induced silencing complex (RISC), a multiprotein complex that mediates gene silencing. One of the essential proteins in RISC is Argonaute (AGO), which plays a critical role in miRNA-mediated gene silencing. AGO binds the miRNA and helps to facilitate its interaction with target mRNA molecules [18].

- Transcription of miRNA Genes: Most miRNAs are transcribed by RNA polymerase II (Pol II) as primary miRNAs (pri-miRNAs), several kilobases long and capped, polyadenylated, and structured with stem–loop formations. Some miRNAs, however, are transcribed by RNA polymerase III. These pri-miRNAs can originate from independent miRNA genes, protein-coding genes’ introns, or polycistronic clusters containing multiple miRNA sequences [19];
- Nuclear Processing of pri-miRNA: Once transcribed, pri-miRNAs undergo processing within the nucleus. A microprocessor complex, composed of the RNase III enzyme Drosha and its cofactor DiGeorge syndrome critical region gene 8 (DGCR8), cleaves the pri-miRNA at the stem–loop region, releasing a shorter precursor miRNA (pre-miRNA) of approximately 70 nucleotides. This cleavage step is crucial for defining the miRNA’s 5′ and 3′ ends [20];
- Nuclear Export of pre-miRNA: The pre-miRNA is then exported from the nucleus to the cytoplasm. Exportin-5, a Ran-GTP-dependent nuclear transport receptor, recognizes the double-stranded stem structure of the pre-miRNA and facilitates its export across the nuclear membrane. The high-affinity interaction between Exportin-5 and pre-miRNA ensures that only properly processed pre-miRNAs are transported out of the nucleus [21];
- Cytoplasmic Processing of pre-miRNA: Once in the cytoplasm, the pre-miRNA is further processed by the RNase III enzyme Dicer, which cleaves the loop structure of the pre-miRNA, yielding a miRNA duplex of about 22 nucleotides. Dicer partners with the trans-activator RNA-binding protein (TRBP) and Argonaute (AGO) proteins to form the RNA-induced silencing complex (RISC). One strand of the miRNA duplex, the guide strand, is predominantly loaded onto AGO proteins in RISC, while the complementary passenger strand is degraded [22];
- Functional Maturation and Targeting: The mature miRNA-RISC complex functions in gene silencing. The miRNA guides RISC to target mRNAs, where it typically binds to the 3′ UTRs of target transcripts through imperfect base-pairing. If the complementarity is high, this binding leads to either translational repression or mRNA cleavage. The extent of complementarity between the miRNA and its target determines the mode of the gene silencing [6].

**Figure 3:**
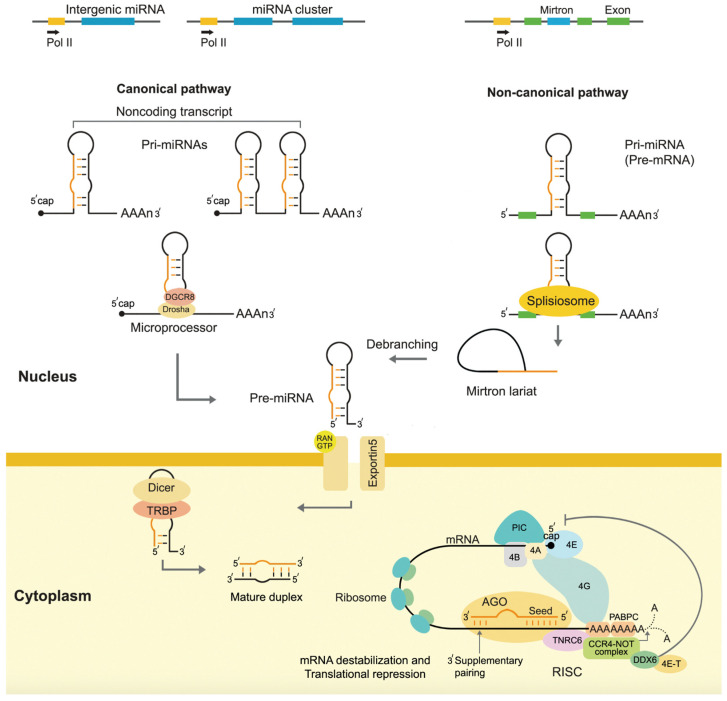
Overview of the miRNA biogenesis. In the canonical pathway in the nucleus, pri-miRNAs are cleaved into pre-miRNAs by Drosha. Pre-miRNAs are exported to the cytoplasm by Exportin 5. In the cytoplasm, pre-miRNAs are cleaved into small dsRNAs by Dicer. Then, RISC mediates the recognition of the mRNA to be targeted. In the non-canonical pathway (Mirtron), Drosha cleavage is substituted with splicing. Pri-miRNAs (pre-mRNAs) are processed to pre-miRNAs by the spliceosome machinery and debranching enzyme to generate double-stranded loop structures, like regular miRNAs. Subsequently, the RNA product of this splicing adopts a pre-miRNA-like form and is transferred to the cytoplasm by Exportin 5 to continue with the canonical pathway (https://en.wikipedia.org/wiki/File:MiRNA-biogenesis.jpg (accessed on 12 October 2024)).

Briefly, the biogenesis of miRNAs involves transcription by Pol II, nuclear processing by Drosha, export by Exportin-5, cytoplasmic maturation by Dicer, and final loading into RISC, where miRNAs exert their gene-silencing effects. This regulatory pathway is crucial for various biological processes, including development, cell differentiation, and disease mechanisms (Figure 3).

### 4.1. Biogenesis in Plants

MiRNA biogenesis in plants differs from animal biogenesis in the nuclear-processing and export steps. Instead of being cleaved by two different enzymes, once inside and once outside the nucleus, both cleavages of the plant miRNA are performed by a Dicer homolog called Dicer-like1 (DL1). DL1 is expressed only in plant cells’ nuclei, indicating that both reactions occur inside the nucleus. Before plant miRNA:miRNA* duplexes are transported out of the nucleus, their 3′ overhangs are methylated by an RNA methyltransferase protein called Hua-Enhancer1 (HEN1). The duplexes are then transported out of the nucleus to the cytoplasm by a protein called Hasty [23], an Exportin 5 homolog, where they disassemble, and the mature miRNA is incorporated into the RISC [24]. 

### 4.2. Evolution

MiRNAs are well conserved in plants and animals and are considered as a vital and evolutionarily ancient component of gene regulation. Although core components of the microRNA pathway are conserved between plants and animals, miRNA repertoires in the two kingdoms appear to have emerged independently with different primary modes of action [25]. 

MicroRNAs are useful phylogenetic markers because of their low rate of evolution [26]. MicroRNAs’ origin as a regulatory mechanism developed from previous RNAi machinery initially used as a defense against exogenous genetic material, such as viruses [27]. Their origin may have permitted the development of morphological innovation and, by making gene expression more specific and ‘fine-tunable’, allowed for the genesis of complex organs [28] and, perhaps ultimately, complex life. Rapid bursts of morphological innovation are generally associated with a high rate of microRNA accumulation [28]. 

New microRNAs are created in multiple ways. Novel microRNAs can originate from the random formation of hairpins in “noncoding” sections of DNA (i.e., introns or intergenic regions) but also by the duplication and modification of existing microRNAs [29]. MicroRNAs can also form from inverted duplications of protein-coding sequences, which allows for creating a foldback hairpin structure [30]. The rate of evolution (i.e., nucleotide substitution) in recently originated microRNAs is comparable to that elsewhere in the noncoding DNA, implying evolution by neutral drift; however, older microRNAs have a much lower rate of change (often less than one substitution per hundred million years), suggesting that once a microRNA gains a function, it undergoes purifying selection [29]. Individual regions within an miRNA gene face different evolutionary pressures, where regions that are vital for processing and functioning have higher levels of conservation [31]. At this point, a microRNA is rarely lost from an animal’s genome [32], although newer microRNAs (thus, presumably non-functional) are frequently lost. In *Arabidopsis thaliana*, the net flux of miRNA genes has been predicted to be between 1.2 and 3.3 genes per million years [33]. This makes them a valuable phylogenetic marker, and they are being looked upon as a possible solution to outstanding phylogenetic problems, such as the relationships of arthropods [34]. On the other hand, in multiple cases, microRNAs correlate poorly with phylogeny, and it is possible that their phylogenetic concordance primarily reflects a limited sampling of microRNAs [35]. 

MicroRNAs feature in the genomes of most eukaryotic organisms, from brown algae to animals [36]. However, the differences in how these microRNAs function and how they are processed suggests that they arose independently in plants and animals [37]. 

## 5. Mechanisms of miRNA Action

In human and animal cells, miRNAs primarily act by destabilizing mRNA that controls gene expression at the posttranscriptional level [38,39]. Dysregulation of miRNAs reflects the state and function of cells and tissues, contributing to dysfunction. Identifying hundreds of extracellular miRNAs in biological fluids highlights their potential as biomarkers.

MiRNAs regulate gene expression by binding to complementary sequences on target mRNAs, leading to mRNA degradation or the inhibition of translation. This posttranscriptional regulation controls various biological processes, from cell development to disease progression. Insights into miRNA production and regulatory mechanisms have expanded our understanding of gene regulation, revealing their role in fine-tuning gene expression.

The degree of complementarity between miRNAs and their target mRNAs mostly determines their regulatory mechanism. In animals, miRNAs typically exhibit partial complementarity, particularly in the “seed region” (nucleotides 2–8), which is crucial for target recognition. Binding to the 3′ untranslated region (3′ UTR) of mRNAs through partial complementarity usually results in translational repression. This process involves the miRNA-RISC complex interfering with the translation machinery, preventing protein production. In some cases, it also leads to mRNA deadenylation and decapping, ultimately causing mRNA degradation [7].

In contrast, miRNAs in plants, and occasionally in animals, can exhibit near-perfect complementarity to target mRNAs. This results in mRNA cleavage and rapid degradation, effectively silencing the gene. Regardless of whether the mechanism involves mRNA degradation or translational inhibition, the outcome is reduced expression of the target [19].

MiRNAs regulate numerous cellular functions by targeting multiple genes. A single miRNA can regulate hundreds of mRNAs, and one mRNA can be targeted by multiple miRNAs, enabling miRNAs to form complex gene regulatory networks. For instance, miR-1 and miR-133, involved in muscle development, perform complementary functions: miR-1 promotes muscle differentiation, while miR-133 enhances the proliferation of muscle progenitor cells. Together, they coordinate muscle growth and development. Similarly, miR-155 plays a critical role in immune responses in the immune system, particularly during inflammation and immune cell activation [40].

Beyond basic gene regulation, miRNAs influence complex processes, such as cellular differentiation, development, and disease progression. In oncogenesis, miRNA dysregulation can activate oncogenes or suppress tumor suppressors, contributing to tumor development and progression [12,41,42].

MRNA molecules act by one or more processes [2,38]. These mechanisms of miRNA action are described and assembled in a unified mathematical model as follows [43]:

- Cap-40S initiation inhibition;
- 60S ribosomal unit joining inhibition;
- Elongation inhibition;
- Ribosome drop-off (premature termination);
- Co-translational nascent protein degradation;
- Sequestration in P-bodies [44];
- MRNA decay (destabilization) by shortening its poly(A) tail;
- MRNA cleavage, where the mRNA strand is cleaved into two pieces;
- Transcriptional inhibition through microRNA-mediated chromatin reorganization, followed by gene silencing;
- Histone modification and DNA methylation of promoter sites affect target gene expression [45,46].

Discarding these mechanisms using experimental data about stationary reaction rates is often impossible. Nevertheless, they are differentiated in dynamics and have different kinetic signatures [43]. 

### 5.1. RNA-Induced Silencing Complex (RISC)

Dicer is an enzyme in the RNase III family that plays a key role in the biogenesis of small RNAs, like miRNAs and siRNAs. It cleaves double-stranded RNA precursors into shorter double-stranded fragments, which are then processed into functional small RNAs. Dicer specifically processes precursor miRNAs (pre-miRNAs) into mature miRNA duplexes, consisting of the guide (mature miRNA) and the passenger strand. This mature miRNA is then incorporated into the RNA-induced silencing complex (RISC), which guides gene silencing by binding to target mRNAs. 

The mature microRNA [1] becomes a part of the RNA-induced silencing complex (RISC), a critical effector machinery responsible for gene silencing. RISC comprises multiple proteins, with the core being an Argonaute (AGO) protein, which directly binds the mature miRNA. Although Dicer is crucial for miRNA biogenesis, its role within the mature RISC is transient. In the initial stages, Dicer cleaves precursor miRNAs (pre-miRNAs) into mature miRNA duplexes, but it does not remain as a stable component of the active RISC. Instead, the key protein remaining in the active complex is Argonaute, particularly AGO2, which is responsible for mediating the downstream gene-silencing effects [47].

Once the miRNA duplex is generated, one strand, referred to as the guide strand, is loaded onto AGO2, while the other, called the passenger strand, is typically degraded. The guide strand, now bound within AGO2, directs RISC to target mRNAs. Complementarity between the miRNA and the target mRNA guides the RISC complex to specific mRNAs, where AGO2 exerts its gene-silencing function. In perfect or near-perfect complementarity cases, AGO2 can cleave the target mRNA directly, leading to mRNA degradation. Alternatively, if the complementarity is partial, RISC can repress translation or cause mRNA deadenylation and decay by recruiting additional regulatory proteins [48]. Other associated proteins, such as GW182, are crucial for these processes, as they link the RISC complex to the mRNA degradation machinery, including deadenylases and decapping enzymes [48].

### 5.2. Mode of Silencing and Regulatory Loops

Gene silencing may occur either via mRNA degradation or by preventing mRNA from being translated. For example, miR16 contains a sequence complementary to the AU-rich element [49] found in the 3′UTR of many unstable mRNAs, such as TNF alpha or GM-CSF [50]. It has been demonstrated that AGO2 can cleave the mRNA and lead to direct mRNA degradation, given complete complementarity between the miRNA and target mRNA sequence. In the absence of complementarity, silencing is achieved by preventing translation [51]. The relation of miRNA and its target mRNA can be based on the simple negative regulation of a target mRNA. Still, it seems that a common scenario is the use of a “coherent feed-forward loop”, a “mutual negative feedback loop” (also termed as a double negative loop), and a “positive feedback/feed-forward loop”. Some miRNAs work as buffers of random gene expression changes arising from stochastic transcription, translation, and protein stability events. Such regulation is typically achieved by negative feedback loops or incoherent feed-forward loops uncoupling protein output from mRNA transcription.

### 5.3. Turnover

The turnover of mature miRNA is needed for rapid changes in miRNA expression profiles. During miRNA maturation in the cytoplasm, uptake by the Argonaute protein is thought to stabilize the guide strand, while the opposite (* or “passenger”) strand is predominantly destroyed. In what has been called a “use it or lose it” strategy, Argonaute may predominantly retain miRNAs with many targets over miRNAs with few or no targets, leading to the degradation of the non-targeting molecules [52]. 

The decay of mature miRNAs in *Caenorhabditis elegans* is mediated by the 5′-to-3′ exoribonuclease XRN2, also known as Rat1p [53]. In plants, SDN (small-RNA-degrading nuclease) family members degrade miRNAs in the opposite (3′-to-5′) direction. Similar enzymes are encoded in animal genomes, but their roles have not been described [52]. 

Several miRNA modifications affect miRNA stability. As indicated by work on the model organism *Arabidopsis thaliana* (thale cress), mature plant miRNAs appear to be stabilized by adding methyl moieties at the 3′ end. The 2′-O-conjugated methyl groups block the addition of uracil (U) residues by uridyl transferase enzymes, a modification that may be associated with miRNA degradation. However, uridylation may also protect some miRNAs; the consequences of this modification are incompletely understood. The uridylation of some animal miRNAs has been reported. Both plant and animal miRNAs may be altered by adding adenine (A) residues to the 3′ end of the miRNA. An extra A added to the end of mammalian miR-122, a liver-enriched miRNA important in hepatitis C, stabilizes the molecule, and plant miRNAs ending with an adenine residue have lower decay rates [52]. 

### 5.4. Cellular Functions

**Figure 4:**
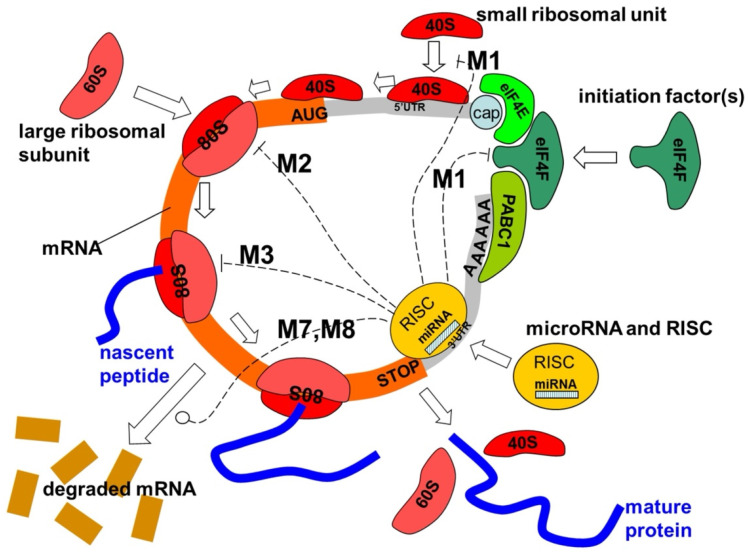
Interaction of microRNA with the protein translation process. Several translation repression mechanisms are shown: (M1) in the initiation process, preventing assembling of the initiation complex or recruiting the 40S ribosomal subunit; (M2) in the ribosome assembly; (M3) in the translation process; (M7, M8) in the degradation of mRNA [43]. The 40S and 60S are light and heavy components of the ribosome, 80S is the assembled ribosome bound to mRNA, eIF4F is a translation initiation factor, PABC1 is the poly-A binding protein, and “cap” is the mRNA cap structure needed for mRNA circularization (which can be the normal m7G-cap or modified A-cap). The initiation of the mRNA can proceed cap independently by recruiting 40S to the IRES (internal ribosome entry site) located in the 5′UTR region. The actual work of RNA silencing is performed by RISC, in which the main catalytic subunit is one of the Argonaute proteins (AGO), and miRNA serves as a template for recognizing specific mRNA sequences (https://commons.wikimedia.org/wiki/File:MiRNA_mechanisms.jpg (accessed on 12 October 2024)).

To regulate genes, an miRNA is complementary to a part of one or more messenger RNAs (mRNAs). Animal miRNAs are usually complementary to a site in the 3′ UTR, whereas plant miRNAs are generally complementary to coding regions of mRNAs [54]. Perfect or near-perfect base pairing with the target RNA promotes the cleavage of the RNA [55]. This is the primary mode of plant miRNAs [56]. In animals, these matchups are imperfect (Figure 4).

For partially complementary microRNAs to recognize their targets, nucleotides 2–7 of the miRNA (its ‘seed region’) [57] must be perfectly complementary [58]. Animal miRNAs inhibit the protein translation of the target mRNA [59], which is present but less common in plants [56]. Partially complementary microRNAs can also speed up deadenylation, causing mRNAs to be degraded sooner [60]. 

Unlike plant microRNAs, animal microRNAs target diverse genes. However, genes involved in functions common to all cells, such as gene expression, have relatively fewer microRNA target sites and seem to be under selection to avoid targeting by miRNAs [61]. There are strong correlations between *ITPR* gene regulations and mir-92 and mir-19 [7]. 

Interactions between microRNAs and complementary sequences in genes, and even pseudogenes that share sequence homology, are considered as a back channel of communication for regulating expression levels between paralogous genes (genes with a similar structure, indicating divergence from a common ancestral gene). Given the name “competing endogenous RNAs” (ceRNAs), these microRNAs bind to “microRNA response elements” in genes and pseudogenes and may provide another explanation for the persistence of noncoding DNA [62]. 

### 5.5. Exosomes

Exosome miRNAs represent a groundbreaking endogenous nanotechnology that leverages the body’s natural cellular communication system to regulate diverse biological processes. Encapsulated within nanoscale extracellular vesicles derived from the endosomal pathway, exosome miRNAs are shielded from enzymatic degradation and efficiently delivered to recipient cells [63]. These miRNAs play critical roles in intercellular communication, mediating gene regulation in immunity modulation, tissue repair, and cancer progression processes, for example, tumor-derived exosomes carrying miR-21 and miR-29a reprogram immune cells, promoting tumor growth and metastasis [64]. In contrast, endothelial-cell-derived exosome miR-126 enhances angiogenesis and vascular repair, showcasing their therapeutic potential in cardiovascular diseases [64].

A distinctive feature of exosome miRNAs is their capacity for targeted delivery, as exosomes travel through bodily fluids and precisely interact with recipient cells via surface receptors, ensuring precise gene modulation. Advances in exosome engineering have paved the way for their use as delivery vehicles for therapeutic miRNAs, enabling innovative treatments for cancer, neurodegenerative diseases, and immune disorders. The natural role of exosome miRNAs as “inner-space” messengers bridges biological systems with therapeutic development, heralding a future where they become central to precision medicine [65]. Furthermore, the exosomal mRNA cargo plays a role in implantation by modulating trophoblast–endometrium adhesion through regulating adhesion/invasion gene expression [66].

The stability of miRNAs in biological fluids is significantly enhanced by their association with exosomes and the Argonaute 2 (AGO2) protein. Urinary miRNAs secreted within exosomes are protected against RNase degradation, enabling their detection, even under harsh conditions, and making them ideal candidates for non-invasive diagnostics [67,68]. MiRNAs associated with AGO2, a key component of the RNA-induced silencing complex (RISC), remain stable outside vesicles because of tight protein binding [69]. This dual stabilization mechanism ensures that urinary miRNAs persist in a functional form, retaining their regulatory and diagnostic potentials. For instance, exosomal miR-21 and miR-29 have been implicated in kidney fibrosis and cancer, emphasizing the clinical relevance of their stabilization [70]. The interplay between exosome packaging and AGO2 binding reflects the sophisticated nature of miRNA-mediated intercellular communication. These insights have spurred the development of diagnostic platforms that exploit the robustness of urinary miRNAs for high-precision disease detection [71].

## 6. Experimental Methods

More than 3000 unique human miRNAs have been recognized and stored in the miRTarBase repository, with a vast, estimated capacity of about five million potential miRNA–target interactions [72]. However, there are issues with the quality of miRNAs. The recognition of miRNAs as a distinct class of regulatory molecules had a transformative effect on molecular biology. For decades, the dominant paradigm in gene regulation focused on transcription factors and promoter regions as the main control points for gene expression [73]. The discovery of miRNAs revealed a layer of posttranscriptional regulation that allowed for cells to fine-tune gene expression more precisely. MiRNAs act by binding to target mRNAs, typically in their 3′ UTR, leading either to mRNA degradation or to the inhibition of translation. This process is highly specific, with each miRNA regulating a wide range of genes, adding complexity to the gene regulatory networks that control cellular functions [74].

The discovery of miRNAs also provided insights into the evolutionary conservation of gene regulatory mechanisms. The fact that miRNAs, like let-7, were conserved from nematodes to humans indicated that miRNA-mediated regulation is a fundamental biological process. Furthermore, miRNAs were found to be involved in a variety of cellular functions, including development, differentiation, cell cycle control, and apoptosis. These small molecules were shown to regulate critical developmental processes, ensuring that cells differentiate and proliferate at the appropriate times and locations. Disruptions in miRNA regulation were linked to various developmental disorders and diseases [5].

MiRNAs are annotated in publicly available databases, as well as the reproducibility of microRNA studies [9]. However, only about 500 human miRNAs represent bona fide miRNAs in the manually curated miRNA gene database MirGeneDB [75]. The exact number continues to increase as new sequencing technologies and computational methods reveal additional miRNAs. Notably, recent studies have identified hundreds of novel miRNAs that are involved in the human brain and other tissues, expanding the known miRNA repertoire [76,77].

While researchers focused on miRNA expression in physiological and pathological processes, various technical variables related to microRNA isolation emerged. The stability of stored miRNA samples has been questioned [78]. MicroRNAs degrade much more easily than mRNAs, partly because of their length and ubiquitously present RNases. This makes it necessary to cool samples on ice and use RNase-free equipment [79]. 

MicroRNA expression can be quantified in a two-step polymerase chain reaction process of modified RT-PCR followed by quantitative PCR. Variations of this method achieve absolute or relative quantification [80]. MiRNAs can also be hybridized to microarrays, slides, or chips with probes to hundreds or thousands of miRNA targets so that relative levels of miRNAs can be determined in different samples [81]. MicroRNAs can be discovered and profiled by high-throughput sequencing methods (microRNA sequencing) [82]. The activity of an miRNA can be experimentally inhibited using a locked nucleic acid (LNA) oligo, a Morpholino oligo [83], or a 2′-O-methyl RNA oligo [84]. A complementary antagomir can silence a specific miRNA. MicroRNA maturation can be inhibited at several points by steric-blocking oligos [85]. A steric-blocking oligo can also block the miRNA target site of an mRNA transcript [86]. LNA or Morpholino probes can be used for the “in situ” detection of miRNA [87]. The locked conformation of LNA results in enhanced hybridization properties and increases sensitivity and selectivity, making it ideal for detecting short miRNAs [88]. 

The high-throughput quantification of miRNAs is error prone for the more significant variance (compared to mRNAs) that comes with methodological problems. MRNA expression is, therefore, often analyzed to check for miRNA effects on their levels [89]. Databases can pair mRNA and miRNA data that predict miRNA targets based on their base sequence [90]. Although this is usually performed after miRNAs of interest have been detected (e.g., because of high expression levels), ideas for analysis tools that integrate mRNA and miRNA expression information have been proposed [91]. 

Just as miRNA is involved in the normal functioning of eukaryotic cells, so has dysregulation of miRNA been associated with disease. A manually curated, publicly available database, miR2Disease, documents known relationships between miRNA dysregulation and human disease [92]. 

## 7. Applications

To use miRNA for inhibiting specific proteins, miRNA mimics, which are synthetic molecules to imitate the functions of endogenous miRNAs, are frequently used to restore the activity of underexpressed miRNAs, promoting the degradation of disease-causing proteins [93]. They are beneficial for conditions where the endogenous miRNA that normally suppresses a harmful protein is downregulated. MiRNAs are also found as extracellular circulating miRNAs [94]. 

Anti-miRs (antagomirs) and chemically modified oligonucleotides that bind to endogenous miRNAs and inhibit their functions are also used. These are used when an overexpressed miRNA promotes the expression of harmful proteins. By inhibiting these miRNAs, anti-miRs prevent the downregulation of tumor suppressor genes or other beneficial proteins [95].

Researchers began exploring the roles of miRNAs in cardiovascular diseases, neurodegenerative disorders, and immune regulation. For example, miR-126 is critical for angiogenesis, the process by which new blood vessels form, and its dysregulation is associated with atherosclerosis and other cardiovascular diseases [96].

### 7.1. Biomarkers

MicroRNAs (miRNAs) have emerged as advanced biomarkers because of their stability in biological fluids, specificity to disease states, and ability to reflect pathophysiological changes. Found in plasma, urine, saliva, and other fluids, they are often encapsulated in exosomes or bound to proteins, like Argonaute, which protect them from degradation, making them ideal for non-invasive diagnostic testing [97]. For example, miR-21 is a well-known biomarker for breast, colon, and lung cancers, with elevated levels correlating with tumor presence, progression, and poor prognosis [98]. Circulating miR-126 and miR-499 are sensitive indicators of myocardial injury and endothelial dysfunction, aiding the early diagnosis of heart attacks [99], while miR-122 and miR-192 are valuable for detecting liver diseases, like hepatitis and drug-induced liver injury, because of their liver-specific expressions [100]. Additionally, miRNAs, such as miR-155, serve as markers of treatment efficacy, with declining levels during chemotherapy in lymphoma patients signaling a therapeutic response [101]. Advances in high-throughput sequencing and qPCR technologies have further enhanced miRNA profiling in clinical settings, underscoring their utility in early disease detection, personalized medicine, and therapeutic monitoring.

A promising application of miRNAs is the development of synthetic miRNA circuits acting as biosensors within cells. These circuits detect cellular changes, such as disease indicators, and modulate miRNA activity to facilitate a controlled therapeutic response. Such circuits hold particular promise in cancer treatment, where they could target tumor cells while sparing healthy tissue [102]. MiRNAs in blood, saliva, and urine remain stable in circulation, serving as ideal candidates for non-invasive diagnostics. Dysregulated miRNAs indicate tissue conditions, with miR-155 linked to multiple cancers and miR-126 associated with cardiovascular diagnostics because of its role in atherosclerosis.

The non-invasive nature of miRNA-based liquid biopsies offers an alternative to traditional diagnostic methods that often require invasive procedures. Liquid biopsies enable early disease identification, treatment monitoring, and recurrence surveillance by assessing alterations in circulating miRNAs, such as miR-21, in oncology. Circulating miRNAs in blood and cerebrospinal fluid also function as biomarkers for disorders [94,103].

This approach is beneficial for detecting tumors, which recurrence or metastasis can precede findings from conventional imaging techniques.

Future advancements in miRNA research may lead to disease-specific diagnostic panels that enhance patient outcomes through personalized medicine. By identifying distinct miRNA expression profiles, clinicians could offer tailored diagnostic and prognostic insights, facilitating timely interventions and customized treatments. Integrating miRNAs with other molecular markers, such as circulating tumor DNA (ctDNA), could further improve the specificity and sensitivity of diagnostic assays [104].

The potential of synthetic miRNA circuits extends beyond diagnostics to gene regulation, where these circuits can detect specific illness indicators and modulate miRNA activity for a promising highly targeted therapeutic specifically targeting cancer, offering the precision targeting of tumor cells while minimizing damage to healthy tissues [105]. These innovations herald a new era in molecular diagnostics and therapeutic strategies, leveraging miRNAs’ unique stability, specificity, and regulatory capabilities.

### 7.2. Cancer

The involvement of miRNAs in cancer is one of the most extensively researched areas in miRNA studies, given their critical roles in regulating cell proliferation, differentiation, and survival. The first human disease linked to miRNA dysregulation was chronic lymphocytic leukemia [106], and since then, numerous miRNAs associated with cancer have been termed as “oncomiRs” [107]. In cancerous B-cells, miRNAs are integral to B-cell receptor (BCR) signaling, motility, adhesion, interactions within immunological niches, immunoglobulin production, and class switching. They also influence the maturation and development of various B-cell types, including pre-B, marginal zone, follicular, plasma, and memory B-cells [108].

MiRNAs are valuable prognostic tools in cancer. For instance, diminished levels of miR-324a in non-small cell lung cancer (NSCLC) and altered levels of miR-185 and miR-133b in colorectal cancer are associated with poor survival outcomes and metastasis [109,110]. MiRNAs also correlate with histological subtypes of colorectal cancer, with miR-205 and miR-373 being elevated in mucin-producing tumors and those linked to ulcerative colitis but not in non-mucinous adenocarcinomas [111]. In hepatocellular carcinoma, miR-21 promotes tumor cell proliferation by interacting with the MAP2K3 tumor suppressor gene [112]. Circulating miRNAs (cimiRNAs), such as plasma miR-21, miR-494, and miR-1973, are stable in blood and serve as potential biomarkers for monitoring disease responses in conditions like classical Hodgkin lymphoma [113]. These biomarkers enable clinicians to evaluate disease progression, interpret imaging results, and detect relapses.

MiRNAs are also critical to cancer progression by regulating DNA repair genes. Cancer often results from mutations caused by DNA replication errors or repair deficiencies, and miRNAs modulate numerous genes involved in DNA repair pathways [114,115]. Germline mutations in DNA repair genes, regulated by miRNAs, account for 2–5% of colorectal cancer cases [116]. Dysregulated miRNA expression contributes to DNA repair defects, which can be causal factors in malignancies. Proteins, such as HMGA1a, HMGA1b, and HMGA2, frequently overexpressed in cancers, like thyroid, colorectal, and ovarian carcinomas, are modulated by miRNAs and play roles in tumorigenesis [107]. Moreover, SNPs can alter miRNA binding to 3′ UTRs, as seen in hsa-mir181a and hsa-mir181b interactions with the CDON tumor suppressor gene [117].

Therapeutically, miRNAs are targeted using two main strategies: restoring tumor-suppressive miRNAs or inhibiting oncomiRs. For example, miR-34, a prominent tumor suppressor often downregulated in cancers like lung and pancreatic malignancies, inspired the development of MRX34, a miRNA mimic. MRX34 demonstrated potential in promoting apoptosis and reducing tumor growth but faced setbacks in clinical trials because of immune-related adverse effects [93,94]. Conversely, tumor-suppressor miRNAs, such as let-7, which targets oncogenes like RAS, MYC, and HMGA2, are frequently downregulated in cancer. Low let-7 levels are linked to poor prognoses in malignancies, such as lung cancer, underscoring their therapeutic relevance [118]. Restoring tumor-suppressive miRNAs and inhibiting oncomiRs are emerging therapeutic approaches with significant potential.

MiRNAs also serve as non-invasive biomarkers for cancer diagnosis and prognosis. Elevated miR-155 levels in blood are associated with unfavorable outcomes in lymphoma and other malignancies [119]. Liquid biopsies leveraging circulating miRNAs in blood and urine offer non-invasive cancer screening and disease monitoring methods. Combining miRNA-based diagnostics with different molecular markers, such as circulating tumor DNA (ctDNA), could enhance cancer detection’s sensitivity and specificity.

Despite setbacks in clinical applications, miRNA therapeutics continue to hold promise. The challenges in delivering miRNA mimics, minimizing immune responses, and achieving effective systemic distribution underscore the need for improved delivery systems and therapeutic strategies. The pioneering work with MRX34 highlights the complexities of miRNA therapies while emphasizing their potential to transform cancer treatment [120].

### 7.3. Cardiovascular Diseases

MicroRNAs (miRNAs) are pivotal in regulating cardiomyocyte survival and death and central to the pathogenesis of cardiovascular diseases, such as myocardial infarction, heart failure, and ischemia–reperfusion injury. These small, noncoding RNAs modulate key signaling pathways at the posttranscriptional level, orchestrating both pro-survival and pro-apoptotic processes. For instance, muscle-specific miR-1 and miR-133 exhibit opposing roles: miR-1 promotes apoptosis by targeting anti-apoptotic genes, like Bcl-2, while miR-133 inhibits cell death by repressing pro-apoptotic factors, such as caspase-9 [121]. Similarly, miR-21, upregulated during cardiac stress, protects cardiomyocytes by targeting PTEN and activating the Akt signaling pathway to promote cell survival [122], whereas miR-34a downregulates anti-apoptotic proteins, like Bcl-2, contributing to cardiomyocyte death in heart failure [123]. The miR-15 family exacerbates ischemic injury by targeting mitochondrial and cell cycle-related genes during reperfusion [123]. These findings highlight the dual regulatory roles of miRNAs and their potential as therapeutic targets. Emerging therapies, such as miRNA mimics and inhibitors (antagomirs), offer strategies to modulate miRNA activity, mitigate cardiomyocyte loss, and enhance cardiac repair.

In addition to their role in the heart, miRNAs are significant in cerebrovascular conditions, like stroke. Stroke, one of the leading causes of death and disability in the U.S., is predominantly ischemic, resulting from arterial blockages that prevent oxygen and nutrient delivery to the brain [124]. MiRNAs are involved in posttranscriptional gene silencing in stroke pathogenesis, targeting inflammatory, angiogenesis, and apoptotic pathways [125]. For example, miR-126, a critical regulator of endothelial cell functions, promotes angiogenesis by targeting the PI3K-Akt pathway. Its reduced expression is associated with atherosclerosis and coronary artery disease, contributing to impaired blood vessel growth and ischemic events. Therapeutic strategies to restore miR-126 expression show promise in promoting vascular repair and reducing ischemic risks [12,124].

MiR-1 and miR-133 also play essential roles in heart muscle development and function. MiR-1 facilitates cardiomyocyte differentiation, while miR-133 supports their proliferation. Dysregulation of these miRNAs is linked to cardiac hypertrophy and heart failure, where improper heart muscle growth and function occur. Restoring the balance of miR-1 and miR-133 in animal models improves cardiac function and reduces scar formation after heart attacks, offering therapeutic potential for heart disease [126]. MiR-133, in particular, has shown promise in enhancing heart muscle regeneration and reducing scar tissue following myocardial infarction, making it a target for preventing heart failure after cardiac injury.

The versatility of miRNAs in cardiovascular disease extends to their roles in angiogenesis, cardiac remodeling, and response to injury. Reduced miR-126 levels, for instance, are consistently linked to vascular diseases, such as atherosclerosis and coronary artery disease, emphasizing its importance in endothelial cell function and vascular integrity. Research into restoring miR-126 expression aims to improve angiogenesis and promote tissue regeneration in ischemic heart disease and myocardial infarction [127].

Overall, miRNAs represent critical regulators in cardiovascular and cerebrovascular health. Their influences on pathways such as angiogenesis, apoptosis, and cardiomyocyte proliferation underscore their therapeutic potential. By restoring or inhibiting specific miRNAs, researchers aim to harness these molecules for treatments that improve outcomes in conditions like heart failure, stroke, and coronary artery disease. These insights shape innovative approaches for mitigating injury and promoting repair in cardiovascular medicine.

### 7.4. Neurodegenerative Diseases

In the nervous system, miRNAs regulate neuronal differentiation, synaptic plasticity, and survival. Dysregulation of miRNAs has been linked to neurodegenerative diseases, such as Alzheimer’s disease, Parkinson’s disease, and Huntington’s disease. MiR-124 is one of the most abundant miRNAs in the brain and plays critical roles in maintaining neuronal identity and promoting synaptic function. In neurodegenerative diseases, miR-124 is often downregulated, leading to neuronal dysfunction and synaptic loss. Restoring miR-124 expression in animal models of neurodegeneration has shown promise in protecting neurons from degeneration and improving cognitive function [128].

In Alzheimer’s disease, miRNAs, such as miR-29 and miR-146a, regulate amyloid-beta production. This toxic protein accumulates in the brains of Alzheimer’s patients. MiR-29 suppresses the expression of beta-secretase, an enzyme involved in amyloid-beta production. Reduced levels of miR-29 in Alzheimer’s patients are associated with increased amyloid-beta accumulation, suggesting that restoring the miR-29 function could help to slow disease progression. Similarly, miR-146a modulates the brain’s inflammatory response, which is often dysregulated in neurodegenerative diseases. Targeting miRNAs involved in inflammation and amyloid-beta production offers a promising therapeutic approach for treating Alzheimer’s disease [129].

### 7.5. Autoimmune Diseases

In the immune system, miRNAs play a central role in regulating inflammation and immune responses by controlling the production of cytokines and other immune-related proteins. MiR-146a and miR-155 are particularly significant in this context. MiR-146a acts as a negative regulator of inflammation by targeting key molecules in the NF-κB signaling pathway, which is activated during immune responses. By limiting excessive inflammatory signaling, miR-146a protects tissues from chronic inflammatory damage. Dysregulation of miR-146a is associated with chronic inflammatory conditions, such as rheumatoid arthritis and lupus, where unchecked inflammation leads to tissue damage. Therapeutic strategies that enhance miR-146a expression have shown promise in reducing inflammation in animal models of autoimmune diseases [7,130].

Conversely, miR-155 promotes immune cell activation and differentiation, including T-cells and macrophages, enhancing the immune response when necessary. However, miR-155 is often upregulated in autoimmune diseases, such as multiple sclerosis and inflammatory bowel disease, which drive overactive immune responses. Inhibiting miR-155 in animal models has reduced disease severity, making it a potential therapeutic target for modulating immune responses in autoimmune conditions [119].

In neurodegenerative diseases, miRNAs are increasingly recognized for their roles in regulating neuronal function and synaptic plasticity. For instance, miR-124 is highly expressed in neurons, maintaining neuronal identity and supporting synaptic function. Dysregulation of miR-124 and other miRNAs contributes to neuronal loss and synaptic dysfunction in conditions such as Alzheimer’s disease, Parkinson’s disease, and amyotrophic lateral sclerosis (ALS). In Alzheimer’s disease, miRNAs, such as miR-29 and miR-146a, are critical in regulating amyloid-beta production and inflammation. MiR-29 inhibits beta-secretase, the enzyme responsible for producing amyloid-beta plaques, which accumulation is a hallmark of Alzheimer’s disease. Restoring the miR-29 function may help to reduce amyloid-beta levels and slow disease progression. Similarly, miR-146a regulates the brain’s inflammatory response, which becomes dysregulated in Alzheimer’s disease, contributing to disease pathology. Targeting these miRNAs offers therapeutic opportunities to address the underlying causes and symptoms of neurodegenerative diseases [129].

Overall, miRNAs, such as miR-146a and miR-155, are critical regulators of immune balance, while miR-124 and miR-29 contribute to neuronal health. Their dysregulation in autoimmune and neurodegenerative diseases underscores their importance as therapeutic targets. Research into miRNA modulation continues to highlight their potentials in managing inflammation, immune overactivation, and neurodegeneration.

### 7.6. Viral Infections

MiRNAs have shown significant potential as therapeutic agents for viral infections, particularly for viruses that exploit host miRNAs for their replication. A notable example is miR-122, a liver-specific miRNA essential for hepatitis C virus (HCV) replication. Miravirsen, an antagomir targeting miR-122, was among the first miRNA-based therapies to enter clinical trials for viral infections. By inhibiting miR-122, miravirsen reduces viral replication and lowers the viral load in HCV-infected patients, offering a novel approach for treating viral infections without directly targeting the virus, thereby minimizing the risk of drug resistance [131].

Similarly, miRNAs are being explored for their potential to inhibit HIV replication. Specific host miRNAs, such as miR-29, can target and degrade HIV transcripts, thereby reducing viral replication. Strategies to enhance the expressions of these host miRNAs or deliver synthetic miRNAs that mimic their functions are under investigation and to develop antiviral therapies that control HIV infection and limit the emergence of drug-resistant strains [132].

Viral miRNAs also play a crucial role in regulating gene expression in both viral and host genes to benefit the virus. These miRNAs are integral to host–virus interactions and the pathogenesis of viral diseases. For example, human herpesvirus-6 is believed to regulate the expression of transcription activators through its viral miRNAs, further illustrating the complex roles miRNAs play in viral infections [4,133].

Although challenges, such as delivery and stability, remain for miRNA-based therapeutics, ongoing advancements in delivery technologies hold promise for improving the feasibility and efficacy of these treatments. Researchers are uncovering innovative strategies to combat viral diseases and reduce drug resistance by targeting host–virus interaction mechanisms through miRNAs.

### 7.7. Alcoholism

The vital role of miRNAs in gene expression is significant to addiction, specifically alcoholism [134]. Chronic alcohol abuse results in persistent changes in brain function, mediated in part by alterations in gene expression [134]. MiRNAs’ global regulation of many downstream genes is deemed as significant regarding the reorganization of synaptic connections or long-term neural adaptations involving behavioral change from alcohol consumption to withdrawal and dependence [135]. Up to 35 different miRNAs are altered in the alcoholic post-mortem brain, all targeting genes that regulate the cell cycle, apoptosis, cell adhesion, nervous system development, and cell signaling [134]. Altered miRNA levels were found in the medial prefrontal cortex of alcohol-dependent mice, suggesting the roles of miRNA in orchestrating translational imbalances and the creation of differentially expressed proteins within an area of the brain where complex cognitive behavior and decision-making likely originate [136]. 

MiRNAs can be either upregulated or downregulated in response to chronic alcohol use. MiR-206 expression increased in the prefrontal cortex of alcohol-dependent rats, targeting the transcription factor brain-derived neurotrophic factor (BDNF) and, ultimately, reducing its expression. BDNF plays a critical role in the formation and maturation of new neurons and synapses, suggesting a possible implication in synapse growth/synaptic plasticity in alcohol abusers [137]. MiR-155, important in regulating alcohol-induced neuroinflammation responses, was upregulated, suggesting the roles of microglia and inflammatory cytokines in alcohol pathophysiology [138]. Downregulation of miR-382 was found in the nucleus accumbens, a structure in the basal forebrain, which regulates feelings of reward that power motivational habits. MiR-382 is the target for dopamine receptor D1 (DRD1), and its overexpression results in the upregulation of DRD1 and delta fosB, a transcription factor that activates a series of transcription events in the nucleus accumbens that ultimately result in addictive behaviors [139]. Alternatively, overexpressing miR-382 resulted in attenuated drinking and DRD1 inhibition and delta fosB upregulation in rat models of alcoholism, demonstrating the possibility for using miRNA-targeted pharmaceuticals in treatments [139]. 

### 7.8. Aging

MiRNAs play pivotal roles in regulating lifespan, metabolism, and aging by modulating gene expression posttranscriptionally. Proteins associated with aging processes, such as those in the insulin-like growth factor (IGF) pathway and oxidative stress response, are often downregulated by miRNAs [140]. For example, miR-34 influences longevity by targeting stress response and mitochondrial function genes, as seen in *Caenorhabditis elegans*, where it modulates insulin/IGF-1 signaling (IIS) and enhances stress resilience [141]. Similarly, miR-29 and miR-181 are involved in mammals’ lipid metabolism and mitochondrial dynamics, linking miRNAs to metabolic health and age-related diseases, like diabetes and obesity [142]. The let-7 family also plays a central role in metabolic programming, regulating glucose homeostasis and adipogenesis by repressing genes, such as IRS2 and HMGA2 [143]. Dysregulation of miRNAs, like miR-146a and miR-21, in aged tissues contributes to inflammation and metabolic disorders, highlighting their roles in age-associated metabolic decline [143]. These insights into the miRNA-mediated control of aging and metabolism suggest potential therapeutic strategies using miRNA mimics or inhibitors to address age-related diseases and enhance lifespan [144]. 

The discovery of lin-4 in *C. elegans* marked a breakthrough in molecular biology, establishing the foundational role of miRNAs in gene regulation. Lin-4, the first identified miRNA, regulates developmental timing by binding to complementary sequences in the 3′ untranslated region (UTR) of lin-14 mRNA, leading to translational repression without degradation. This precise mechanism ensures proper temporal progression of developmental events in *C. elegans*, demonstrating the fine-tuning capabilities of miRNAs [8]. Beyond its historical significance, lin-4 is a model for understanding miRNA pathways involved in stem cell differentiation, cancer biology, and aging [145]. The lin-4/lin-14 axis exemplifies the elegant simplicity of miRNA regulation, where a single small RNA can orchestrate complex genetic networks, inspiring advancements in miRNA research and therapeutic applications.

Studies of miRNA mutants across species, including *C. elegans*, *Drosophila melanogaster*, and mice, have provided critical insights into miRNA functions in development, physiology, and disease. In *C. elegans*, lin-4 regulated developmental timing by targeting lin-14 mRNA, while let-7 mutants revealed miRNA roles in cell differentiation and longevity [8,145]. In *Drosophila*, miRNA mutants, such as bantam, demonstrated cell proliferation and apoptosis regulation, emphasizing their importance in tissue homeostasis and cancer biology [146]. In mice, the deletion of miR-17~92 disrupted embryonic development, B-cell differentiation, and heart formation, underscoring this cluster’s role in survival and differentiation [147]. Mutants lacking miR-21 or miR-155 highlighted their roles in immunity and stress responses, linking miRNA dysregulation to diseases, like cancer and autoimmunity [148,149]. Across species, miRNAs act as buffers within genetic networks, maintaining robustness against environmental and genetic perturbations and offering insights for miRNA-based therapies.

MiRNA dysregulation is also implicated in aging-related processes, such as inflammation, cellular senescence, and reduced tissue repair. As cells age, they often enter a state of cellular senescence, characterized by the secretion of pro-inflammatory cytokines, growth factors, and proteases, collectively known as the senescence-associated secretory phenotype (SASP) [150]. MiRNA-based therapies could target these aging-related pathways, mitigating the effects of senescence and inflammation. For instance, altered miRNA expression in aging tissues contributes to the accumulation of dysfunctional proteins that disrupt normal cellular processes [151]. By targeting these regulatory networks, miRNA therapies are promising to address age-related metabolic dysfunctions and prolong health span.

Endothelial miRNAs play critical roles in vascular homeostasis and are implicated in various conditions, including cerebrovascular complications of COVID-19 and ischemia with no obstructive coronary artery disease (INOCA) in diabetes patients. MiR-24 has emerged as a critical regulator in COVID-19-related cerebrovascular complications by maintaining the blood–brain barrier’s (BBBs) integrity and mitigating vascular inflammation through the regulation of genes, like endothelin-1 and vascular endothelial growth factor (VEGF) [149]. In severe COVID-19 cases, reduced miR-24 levels correlate with increased endothelial permeability, hypercoagulability, and heightened risks of cerebrovascular events, such as stroke [152]. Experimental models suggest that miR-24 downregulation promotes pro-inflammatory cytokine production, leukocyte adhesion, and endothelial injury through increased ICAM-1 and VCAM-1 expressions. Additionally, miR-24 suppresses oxidative stress by targeting NOX2, which becomes critical during COVID-19-induced oxidative damage [153]. Therapeutic strategies to restore the miR-24 expression, such as miRNA mimics, have shown promise in preclinical studies to reduce inflammation and restore vascular integrity, highlighting its potential as a therapeutic target for COVID-19 cerebrovascular complications. 

In diabetic INOCA patients, endothelial miRNAs, such as miR-126 and miR-92a, are critical to vascular integrity and angiogenesis. MiR-126, typically protective by promoting endothelial repair through VEGF signaling, is downregulated in these patients, exacerbating vascular damage and impairing tissue repair [154]. Conversely, miR-92a, which inhibits endothelial function, is upregulated, aggravating dysfunction by repressing pro-angiogenic factors, like KLF2 and KLF4 [155]. Additional dysregulated miRNAs, including pro-inflammatory miR-21 and miR-146a, contribute to oxidative stress and chronic inflammation, hallmarks of endothelial dysfunction in diabetes [156]. These miRNA alterations impair endothelial function and heighten the risk of microvascular complications and adverse cardiovascular events.

Therapeutic modulation of miRNAs mimics restoring protective miRNAs, like miR-126, or inhibiting harmful ones, like miR-92a, and presents a promising strategy to address endothelial dysfunction in COVID-19 and diabetic INOCA. These findings underscore the pivotal role of endothelial miRNAs in maintaining vascular health and offer innovative targets for therapeutic interventions in these high-risk conditions.

The cumulative insights from miRNA research, spanning developmental biology, metabolic regulation, and aging, highlight their significance as master regulators of biological processes and therapeutic targets for age-related diseases.

These proteins contribute to tissue dysfunction and inflammation in aging tissues.

- miR-146a: This miRNA can inhibit IL-6 and IL-1β, two key inflammatory cytokines upregulated in aged, senescent cells. Targeting these cytokines with miR-146a mimics could reduce inflammation and slow tissue degeneration;
- miR-29: This miRNA can inhibit collagen-degrading enzymes, such as matrix metalloproteinases (MMPs), that are involved in the breakdown of the extracellular matrix in aging tissues. MiR-29 mimics could maintain tissue structure and reduce fibrosis, which becomes more common with age [157];
- miR-375: This miRNA inhibits IGF-1R, a receptor in the IGF pathway that promotes growth and can contribute to age-related diseases, like cancer. Overexpression of miR-375 could reduce IGF-1R activity and help to mitigate these risks [158];
- miR-34a: This miRNA has been shown to suppress p53 and Bcl-2, proteins involved in apoptosis and stress responses, respectively. Modulating the levels of miR-34a may help to balance apoptosis in aging cells, potentially reducing unwanted cell death in key tissues, like the brain or heart [159];
- miR-103/107: These miRNAs target caveolin-1, a regulator of insulin signaling. Inhibiting these miRNAs could enhance insulin sensitivity, which typically decreases with age, helping to prevent or manage metabolic diseases [153].

### 7.9. Obesity

MiRNAs play critical roles in regulating the differentiation of stem cell progenitors into adipocytes, with significant implications for understanding adipogenesis and potential obesity treatments of adipocytes [160]. Studies using the immortalized human-bone-marrow-derived stromal cell line hMSC-Tert20 have shown that decreased expressions of miR-155, miR-221, and miR-222 occur during adipogenic programming in both immortalized and primary hMSCs, indicating their role as negative regulators of differentiation [161]. Conversely, ectopic expression of these miRNAs significantly inhibits adipogenesis. It represses the induction of critical adipogenic regulators, such as PPARγ and CCAAT/enhancer-binding protein alpha (CEBPA), providing a pathway for potential genetic obesity therapies [162]. 

Another critical group of miRNAs involved in metabolic regulation is the let-7 family, which has been linked to insulin resistance, obesity, and diabetes. Let-7 levels increase in human tissues during aging [163]. Experimental studies have shown that the ectopic overexpression of let-7 in mice mimics accelerated aging, resulting in insulin resistance and higher propensities for diet-induced obesity and diabetes [164]. Conversely, the inhibition of let-7, using let-7-specific antagomirs, enhances insulin sensitivity and provides remarkable resistance to high-fat-diet-induced obesity and diabetes. Furthermore, let-7 inhibition prevents and can reverse and cure obesity and type 2 diabetes [165]. These findings highlight the therapeutic potential of targeting let-7 to develop innovative treatments for metabolic disorders, such as obesity and diabetes.

### 7.10. Hemostasis

Under normal physiological conditions, miRNAs maintain cellular homeostasis by regulating gene expression in critical processes, like development and cell differentiation. MiR-1 and miR-133, for example, are essential regulators of muscle development. MiR-1 promotes the differentiation of muscle progenitor cells into myocytes, while miR-133 [166] enhances the proliferation of muscle progenitor cells, ensuring a balance between muscle growth and differentiation. These miRNAs are essential for proper muscle formation during the development and regeneration of muscle tissue after injury [6].

MiRNAs also regulate complex enzymatic cascades, including the hemostatic blood coagulation system [161]. Large-scale studies of functional miRNA targeting have recently uncovered rational therapeutic targets in the hemostatic system [167]. They have been directly linked to calcium homeostasis in the endoplasmic reticulum, which is critical in cell differentiation in early development [168]. 

Metabolic regulation is another area where miRNAs are critical. MiR-122, for instance, is highly expressed in the liver and regulates cholesterol and fatty acid metabolism [169]. MiR-122’s involvement in lipid homeostasis underscores its importance in maintaining metabolic health. Dysregulation of miR-122 can lead to metabolic disorders, such as non-alcoholic fatty liver disease (NAFLD) and hyperlipidemia, further emphasizing the role of miRNAs in normal physiology [170].

### 7.11. Others

Furthermore, the growing evidence linking miRNAs to human diseases has spurred interest in developing miRNA-based diagnostics and therapeutics [93]. MiRNA technology is actively developed, leading to many novel applications, such as combining miRNA therapies with treatments like chemotherapy, immunotherapy, or gene therapy, which is an emerging trend [171]. MiRNA modulation can enhance the efficacy of traditional treatments by sensitizing cancer cells to chemotherapy or reducing resistance to drugs. Each database serves different purposes, ranging from miRNA sequence information to prediction and validation of miRNA targets, disease interactions, and more [172]. 

A mutation in the seed region of miR-96 causes hereditary progressive hearing loss [173]. A mutation in the seed region of miR-184 causes hereditary keratoconus with an anterior polar cataract [174]. Deletion of the miR-17~92 cluster causes skeletal and growth defects [175]. 

Moreover, miRNAs, such as miR-183/96/182, play crucial roles in the circadian rhythm [176]. 

### 7.12. Plants

MiRNAs are critical regulators of many developmental, homeostatic, and immune processes in plants [177]; their roles in plant development include shoot–apical meristem development, leaf growth, flower formation, seed production, and root expansion [178]. In addition, they play complex roles in responses to various abiotic stresses, comprising heat stress, low-temperature stress, drought stress, light stress, or gamma radiation exposure [177]. 

### 7.13. MiRNAs in Precision Medicine

Personalized or precision medicine aims to tailor medical treatments to individual patients based on their genetic, environmental, and lifestyle factors. MiRNAs are well suited for integration into precision medicine because they play critical roles in regulating gene expression and can provide insights into a person’s unique genetic profile. As miRNA sequencing technologies improve, it is becoming easier to identify miRNA expression patterns that correlate with specific diseases or responses to therapy. For example, patients with particular miRNA signatures may respond better to specific cancer treatments, while others may require alternative approaches based on their miRNA profiles [179].

Integrating miRNA data into precision medicine frameworks could enable tailored therapies that address the specific molecular abnormalities driving a patient’s disease. In cancer, this could mean identifying oncomiRs upregulated in a particular tumor and using antagomirs to inhibit these miRNAs, thereby halting cancer progression [180]. Similarly, miRNAs regulating vascular health or inflammation could be targeted in cardiovascular disease to prevent disease progression or improve outcomes following events like heart attacks. Personalized miRNA therapies could also reduce the likelihood of adverse drug reactions, as treatments would be based on the patient’s miRNA profile, leading to more effective and safer interventions.

### 7.14. MiRNAs in Regenerative Medicine

As research into miRNAs advances, their application in regenerative medicine is expected to grow significantly. MiRNAs have already been shown to promote differentiation into specific cell types, making them promising tools for tissue regeneration and repair. For instance, miR-375 has been used to induce stem cells to differentiate into insulin-producing beta cells, offering potential therapies for diabetes [20]. Similarly, miR-1 and miR-133 are involved in cardiac muscle regeneration, and manipulating these miRNAs could help to repair heart tissue after injury [181].

In the future, miRNA-based therapies could be used to engineer tissues and organs for transplantation, offering solutions for patients with organ failure or severe injuries. By guiding the differentiation of pluripotent stem cells into functional tissues using miRNAs, researchers could develop personalized tissues that are genetically matched to the patient, reducing the risk of rejection and improving the success of transplants. This approach holds significant potential for the treatment of diseases, like heart failure, diabetes, and neurodegenerative disorders, where organ damage is currently irreversible [182].

### 7.15. Synthetic Biology and miRNA-Based Therapeutics

Synthetic biology is expected to play a critical role in the future of miRNA-based therapeutics, especially in designing custom miRNA circuits [183]. These circuits could function as biosensors to detect specific changes within cells, such as shifts in the expression of disease-associated genes. Once activated, miRNA circuits could trigger precise therapeutic responses, making them ideal for targeted cancer therapies or regenerative medicine. Using miRNAs as molecular switches, these synthetic constructs would enable highly controlled interventions, minimizing the risk of off-target effects that often complicate current therapeutic approaches. Moreover, researchers are investigating the possibility of developing synthetic miRNA circuits that can function as biosensors within cells, responding to changes in the cellular environment and regulating gene expression accordingly. These circuits could provide highly controlled therapeutic responses, activating only when specific disease markers are present and deactivating once the therapeutic goal has been achieved [184]. This synthetic biology approach represents an exciting frontier in miRNA research, with potential applications in regenerative medicine, cancer therapy, and chronic disease management.

For instance, synthetic miRNA circuits in cancer therapy could be designed to detect the expression of oncogenes or specific tumor markers [185]. Upon recognizing these markers, the circuit could activate miRNAs that suppress tumor growth or induce apoptosis in cancer cells while sparing healthy tissues. This level of specificity could drastically improve the outcomes of miRNA-based cancer therapies, reducing side effects and enhancing the treatment efficacy.

### 7.16. MiRNAs in Gene Editing

The discovery of miRNAs and their regulatory roles in gene expression have unveiled new therapeutic possibilities [17]. MiRNAs are pivotal in numerous cellular processes, such as differentiation, proliferation, apoptosis, and metabolism. Dysregulation of miRNAs is implicated in diverse conditions, including cancer, cardiovascular diseases, neurodegenerative disorders, viral infections, and inflammation. Targeting miRNAs through therapeutic approaches, like miRNA mimics and antagomirs (anti-miRNA oligonucleotides), enables the restoration of regular gene expression or inhibition of harmful miRNA activity [186]. These small RNA molecules also serve as valuable biomarkers for disease detection and promising tools for addressing complex diseases [187]. 

Emerging technologies, like CRISPR-Cas9, complement miRNA therapies by offering gene-editing capabilities while miRNAs regulate posttranscriptional gene expression, ensuring appropriate levels of gene activity. This combination can enhance gene therapy precision, reducing off-target effects and complications from overexpression. Researchers are exploring methods to integrate CRISPR and miRNA-based regulation to optimize therapeutic outcomes [188].

Advanced delivery systems, including lipid nanoparticles (LNPs), viral vectors, and exosome-based platforms, enhance miRNA-based drugs’ stability, specificity, and tissue targeting. These technologies protect miRNAs from degradation and improve the delivery efficacy. For example, LNPs deliver miR-34 mimics in cancer therapies, while exosome-based systems show promise in targeting specific cell types without off-target effects. Chemical modifications, such as locked nucleic acids and 2′-O-methylation, further enhance the stability and binding affinity of miRNA mimics and antagomirs, reducing off-target risks and increasing therapeutic precision [188,189,190,191]. As innovations in delivery and chemical modification continue to evolve, the safety and efficacy of miRNA therapies are poised for significant advancements, paving the way for broader clinical application.

### 7.17. Tissue-Specific miRNA

Different tissues have distinct miRNAs that regulate tissue-specific processes, such as cell differentiation, growth, metabolism, and homeostasis. These tissue-specific miRNAs are crucial for maintaining the unique functions of each tissue. Below is a list of some of the critical tissue-specific miRNAs, their primary functions, and the tissues they are associated with.

- MiR-1, miR-133, and miR-206 are known as “myo-miRs” and play crucial roles in regulating muscle development (myogenesis), differentiation, and repair [192]. They suppress genes involved in muscle atrophy and promote the growth of new muscle fibers after injury or exercise;
- MiR-1 and miR-133 are also critical in the heart for controlling cardiac muscle differentiation;
- MiR-208a and miR-499 are mainly involved in cardiac contractility and protecting the heart against hypertrophy;
- MiR-122 is liver-specific and is crucial in cholesterol and fatty acid metabolism. It is also essential for liver development and function. MiR-21 and miR-199a are involved in liver fibrosis and hepatocyte proliferation [193];
- MiR-124 is one of the most abundant miRNAs in the brain and is involved in neuronal differentiation and neuroprotection;
- MiR-9 helps to regulate neurogenesis, while miR-132 and miR-128 are essential for synaptic plasticity and cognitive function;
- MiR-143 and miR-103 regulate adipocyte differentiation and insulin sensitivity, making them necessary in fat metabolism and energy homeostasis. MiR-155 is involved in inflammatory regulation within adipose tissue;
- MiR-375 is crucial for insulin secretion and pancreatic beta cell function. It regulates glucose homeostasis and pancreatic cell differentiation, contributing to overall metabolic health;
- MiR-192 and miR-194 are essential for kidney development and maintaining nephron integrity;
- MiR-29 and miR-21 are involved in kidney fibrosis and injury response, playing critical roles in kidney diseases;
- MiR-21 and miR-126 are involved in lung development, inflammation, and repair processes, particularly during fibrosis;
- MiR-29b regulates collagen deposition and is essential in lung fibrotic disorders. MiR-26a, miR-29b, and miR-214 play critical roles in bone development by regulating osteoblast differentiation and bone formation. These miRNAs are targets for promoting bone repair and regeneration;
- MiR-203 and miR-205 regulate keratinocyte differentiation, maintain skin integrity, and promote wound healing. MiR-31 supports epidermal proliferation and repair after injury;
- MiR-150, miR-155, and miR-223 are critical regulators in hematopoietic cells, controlling the differentiation of immune cells and playing roles in inflammation and immune responses;
- MiR-210 and miR-141 regulate placental development and adaptation to hypoxia;
- MiR-517 is involved in trophoblast function and placental growth;
- MiR-34c and miR-449a are involved in spermatogenesis and regulating Sertoli and Leydig cells;
- MiR-202 plays a role in male fertility and testicular development.

### 7.18. Targeted Delivery Using Tissue-Specific Promoters

MiRNAs can bind to target messenger RNA (mRNA) transcripts of protein-coding genes and negatively control their translation or cause mRNA degradation. It is of crucial importance to identify miRNA targets accurately [194]. A comparison of the predictive performances of eighteen in silico algorithms is available [195]. Large-scale studies of functional miRNA targeting suggest that target prediction algorithms can miss many functional miRNAs [196]. 

One practical approach for achieving tissue-specific miRNA delivery is by incorporating tissue-specific promoters. These genetic elements only activate miRNAs’ expression in specific cell types, allowing for localized action. Muscle-specific promoters can be used for miRNA therapies targeting muscle cells (myocytes). Myosin heavy chain (MHC) promoters or muscle creatine kinase (MCK) promoters are known to be highly active only in skeletal muscle cells. Linking the therapeutic miRNA to these promoters can restrict expression in muscle tissues, minimizing off-target effects in other organs [197]. 

Cardiac-specific promoters, like the alpha-myosin heavy chain (α-MHC) promoter or cardiac troponin T promoter, could be employed for heart-targeted miRNA therapies. These promoters ensure that the miRNA is expressed only in cardiomyocytes, the muscle cells of the heart, allowing for miRNA therapies to regulate gene expression only within the heart and not elsewhere [198]. 

Neuron-specific promoters, such as synapsin-1, neurofilament light chain, or GFAP (glial fibrillary acidic protein) for astrocytes, can target brain tissues. These promoters activate gene expression primarily in neurons or glial cells, ensuring the miRNA functions are localized to the central nervous system (CNS). This specificity is critical for treating neurodegenerative diseases, like Alzheimer’s or Parkinson’s diseases [199].

Designing miRNA products that specifically target muscles, the heart, or the brain involves a combination of tissue-specific promoters, ligand-conjugated nanoparticles, cell-penetrating peptides, aptamers, virus-like particles, and CRISPR-based systems [200]. Each approach offers a unique way to direct miRNA therapies to specific tissues, minimizing off-target effects and enhancing the therapeutic efficacy. As technology advances, more sophisticated and precise delivery systems will emerge, paving the way for miRNA-based therapies that can be tailored to individual tissues or disease conditions [201].

#### 7.18.1. Ligand-Conjugated Nanoparticles for Receptor-Mediated Targeting

Nanoparticles are widely used for delivering miRNAs because of their ability to encapsulate nucleic acids and protect them from degradation. Modifying the surface of nanoparticles with ligands (molecules that bind to specific receptors on target cells) makes it possible to achieve receptor-mediated endocytosis, wherein the nanoparticles are taken up only by cells expressing the corresponding receptors [202].

To direct miRNAs to muscle cells, nanoparticles can be functionalized with ligands that bind to receptors uniquely expressed in myocytes. One promising approach is to use ligands that bind to the IGF-1 receptor, which is highly expressed in muscle cells [203]. Folic acid conjugates, or peptides targeting the insulin-like growth factor receptor, can direct miRNA-loaded nanoparticles to muscle tissues, where they can specifically regulate muscle growth, repair, or metabolism [204].

Aptamers (short, single-stranded DNA or RNA molecules that bind to specific proteins) that target cardiac-specific integrins have also been used to deliver therapeutic molecules directly to the heart. This strategy allows for miRNA products to be precisely delivered to the heart while bypassing other tissues [204].

The blood–brain barrier (BBB) is one of the biggest challenges for brain-targeted therapies. To overcome this, miRNA products can be delivered using nanoparticles conjugated with ligands that target receptors expressed on the BBB. For example, nanoparticles functionalized with transferrin or lactoferrin can exploit the receptor-mediated transcytosis pathway to cross the BBB and deliver miRNAs to the brain. Similarly, rabies virus glycoprotein (RVG)-conjugated nanoparticles can bind to the acetylcholine receptor, facilitating miRNA delivery to neurons [205].

#### 7.18.2. Cell-Penetrating Peptides (CPPs)

Cell-penetrating peptides (CPPs) are short peptides that can facilitate the transport of miRNAs across cellular membranes. These peptides can be engineered to include homing sequences that target certain tissues or cells, providing a versatile tool for tissue-specific delivery.

Peptides derived from muscle-specific proteins can target miRNAs in muscle cells. For example, peptides derived from myostatin (a regulator of muscle growth) can be used to direct miRNA-loaded CPPs to muscle tissues, where they can influence muscle regeneration or metabolism [206].

Heart-specific CPPs can be designed by incorporating peptides derived from natriuretic peptides or angiotensin receptors. These receptors are abundant in cardiomyocytes and endothelial cells in the heart. By engineering CPPs that home in on these receptors, miRNA therapies could be directed to the heart, minimizing off-target effects in other organs [207].

For brain-targeted delivery, CPPs can be conjugated with brain-targeting sequences, like TAT (trans-activator of transcription) or RVG (rabies virus glycoprotein) peptides, which facilitate the penetration of miRNAs across the BBB. These CPPs can be linked to miRNA-loaded nanoparticles or oligonucleotides to deliver therapeutic miRNAs directly to neurons or glial cells in the brain [208].

#### 7.18.3. Aptamers for Cell-Specific Targeting

Aptamers are short RNA or DNA molecules that can bind specifically to cell surface proteins. They can be used as targeting agents to direct miRNA products to specific tissues or cell types.

Aptamers targeting myostatin or fibroblast growth factor receptors (FGFRs) can deliver miRNA therapies to muscle cells [209]. Myostatin is a negative regulator of muscle growth, and aptamers that bind to myostatin-expressing cells can target miRNA therapies to enhance muscle regeneration or treat muscular dystrophy.

In heart-targeted therapies, aptamers can be designed to bind to angiotensin II receptors or cardiac-specific integrins. These aptamers can guide miRNA products to cardiomyocytes, where they can modulate gene expression to promote heart regeneration, protect against ischemic injury, or prevent heart failure.

To target the brain, aptamers that bind to receptors expressed on the BBB, such as the low-density lipoprotein-receptor-related protein (LRP) or the nicotinic acetylcholine receptor, can be used. By attaching these aptamers to miRNA-loaded nanoparticles or oligonucleotides, the miRNA product can cross the BBB and reach specific brain cells [210].

#### 7.18.4. Virus-Like Particles (VLPs) for Tissue-Specific Targeting

Virus-like particles (VLPs) are engineered particles that mimic the structure of viruses but lack viral genetic material. VLPs can be used to encapsulate and deliver miRNAs to specific tissues. By modifying the surface of VLPs with tissue-specific targeting ligands, researchers can direct miRNA therapies to specific organs or cells [211].

VLPs can be engineered with surface proteins that bind to receptors on muscle cells, such as the insulin-like growth factor (IGF-1) receptor. These VLPs can deliver miRNA therapies that promote muscle growth or repair by targeting muscle-specific pathways [212].

VLPs can be designed to include cardiotropic proteins that bind to cardiomyocytes for heart-targeted delivery. These VLPs can deliver miRNA products to the heart, where they can modulate gene expression to treat heart diseases, such as heart failure or myocardial infarction [213].

To target the brain, VLPs can be decorated with proteins that allow them to cross the BBB, such as transferrin or apolipoprotein E (ApoE). Once inside the brain, these VLPs can deliver miRNA products to neurons or glial cells, offering a promising strategy for treating neurodegenerative diseases, like Alzheimer’s and Parkinson’s diseases [214].

Making a viral vector specific to a particular tissue involves modifying its components to ensure that it targets and delivers its genetic payload only to cells of the desired tissue. This can be achieved through several strategies, each focusing on enhancing the vector’s ability to recognize and enter specific cell types [215]. 

The viral capsid (in non-enveloped viruses) or envelope (in enveloped viruses) is the outer structure of the virus that interacts with the target cell’s surface receptors. By modifying the viral capsid or envelope, you can direct the viral vector to bind specifically to highly expressed receptors in the target tissue, thereby achieving tissue-specific tropism.

One of the most common ways to make viral vectors tissue-specific is to incorporate ligands that bind to receptors highly expressed on the target tissue but not elsewhere. These ligands can be peptides, antibodies, or receptor-binding proteins specific to a cell surface marker on the target tissue. For targeting muscle cells, ligands that bind to insulin-like growth factor (IGF-1) receptors, which are highly expressed in muscle tissue, can be incorporated into the viral capsid. This would ensure that the viral vector predominantly enters muscle cells. Natriuretic peptides or integrin receptors could be targeted to target cardiomyocytes (heart muscle cells). Viral vectors could be engineered to display aptamers or antibodies that bind to these cardiac-specific surface proteins. For brain targeting, viral vectors can be modified to interact with transferrin or lactoferrin receptors, which are involved in transcytosis across the blood–brain barrier (BBB). Alternatively, rabies virus glycoprotein (RVG) could specifically target neurons by binding to acetylcholine receptors [216].

Pseudo-typing involves replacing one virus’s viral envelope or capsid proteins with those from another virus that naturally targets the desired tissue. This is often used in enveloped viral vectors, such as lentiviral or retroviral vectors. For brain targeting, a lentiviral vector could be pseudo-typed with the glycoprotein from VSV-G (vesicular stomatitis virus), which can facilitate the entry to neurons. Similarly, pseudo-typing with RVG could allow for the viral vector to target neurons more specifically [215].

A highly effective strategy for ensuring tissue specificity is controlling gene expression using tissue-specific promoters. Even if the viral vector enters multiple tissues, the therapeutic gene (or miRNA) will only be expressed in the target tissue where the promoter is active. For muscle-specific targeting, promoters active only in muscle cells, such as muscle creatine kinase (MCK) and myosin heavy chain (MHC) promoters, can be used. Using these promoters, the viral vector will express the therapeutic gene (or miRNA) only in muscle cells, even if the vector reaches other tissues [217].

For cardiac targeting, promoters specific to heart cells can be used, such as alpha-myosin heavy chain (α-MHC) and cardiac troponin T promoters. These promoters ensure the transgene is expressed only in cardiomyocytes, preventing off-target effects in other tissues [198].

To target neurons or glial cells in the brain, a neuron-specific glial fibrillary acidic protein (GFAP) promoter (for astrocytes) can ensure that the viral vector expresses its cargo only in the CNS, reducing the risk of off-target gene expression [218].

Some viral vectors naturally exhibit tissue tropism, predominantly infecting specific tissues. Selecting the appropriate viral vector with a known tropism can increase the likelihood of targeting the desired tissue. AAV vectors are known for their relatively broad tropism, but different AAV serotypes can be selected based on their natural affinity for specific tissues. For example, AAV1 and AAV9 have a natural affinity for skeletal and cardiac muscles. AAV9 is also efficient at crossing the blood–brain barrier, making it suitable for brain targeting. AAV8 has a strong tropism for the liver but can be modified for other tissues with surface modifications [219].

We can enhance tissue-specific targeting by choosing the appropriate AAV serotype, although additional targeting strategies (like capsid modifications or tissue-specific promoters) may still be required for greater specificity.

#### 7.18.5. Lentiviruses and Retroviruses

Lentiviral and retroviral vectors naturally integrate into dividing cells, making them helpful in targeting tissues, like hematopoietic cells, liver cells, or other proliferating tissues. For non-dividing tissues, pseudo-typing or capsid modification can enhance targeting. One of the simplest ways to achieve tissue-specific delivery is through direct injection into or administration to the target tissue. This approach does not require complex viral modifications but limits the application to localized delivery. Viral vectors can be injected directly into muscle tissue, ensuring that most viral particles enter the target cells. This is often used in gene therapy for muscular dystrophy and other muscle-related conditions. For heart targeting, intracoronary or direct injection into the myocardium during surgery ensures the viral vector is delivered directly to the heart tissue. This method is beneficial in cases where systemic delivery might lead to off-target effects. For brain targeting, intracerebroventricular (ICV) injection or intrathecal injection can bypass the blood–brain barrier and deliver the viral vector directly to the cerebrospinal fluid, ensuring that it reaches neurons or glial cells [220].

#### 7.18.6. CRISPR/Cas9 and Aptamers for Enhanced Targeting

Viral vectors can be combined with CRISPR/Cas9 systems or aptamers (small molecules that bind specific cell surface proteins) for precise tissue-specific editing to enhance tissue targeting. CRISPR/Cas9 can be delivered using viral vectors, but the Cas9 enzyme and guide RNA (gRNA) expression can be controlled using tissue-specific promoters. This ensures that the gene-editing activity occurs only in the desired tissue, minimizing off-target effects in other tissues. Aptamers are short oligonucleotides that can bind specifically to proteins on the surface of target cells. Viral vectors can be conjugated with aptamers that bind to tissue-specific receptors, ensuring that the viral vector is taken up only by the target cells. This method offers highly specific targeting, especially for tissues with unique surface markers [221]. 

#### 7.18.7. CRISPR-Based miRNA Activation Systems

A futuristic and highly targeted approach could use CRISPR-based gene activation systems to selectively upregulate or downregulate miRNA expression in specific tissues. By designing tissue-specific guide RNAs (gRNAs) that target particular miRNA loci, CRISPR-based systems could control miRNA levels only in specific cell types [222].

For example, a CRISPR system could be designed to activate miR-206, which is involved in muscle regeneration, only in skeletal muscle cells. The system would rely on muscle-specific gRNAs and tissue-specific promoters to restrict miRNA activation to the muscle, reducing off-target effects elsewhere in the body.

In the heart, CRISPR-based systems could upregulate miRNAs, such as miR-1 and miR-133, which promote cardiomyocyte survival and regeneration. These systems would be activated only in cardiomyocytes, ensuring therapeutic miRNA expression is localized to the heart [222].

## 8. Delivery

Two major strategies are employed when considering the use of miRNA technology for therapeutic purposes: ex vivo manipulation and in vivo administration. Both approaches aim to achieve precise gene regulation while minimizing off-target effects, but each has distinct advantages and limitations [223].

### 8.1. Ex Vivo Manipulation

MicroRNAs (miRNAs) can be synthesized using in vitro transcription (IVT) methods similar to those employed for messenger RNA (mRNA) production. However, miRNA synthesis requires additional steps to ensure the proper folding and processing of the precursor miRNA (pre-miRNA) into its functional form [224].

Ex vivo manipulation involves extracting cells from a patient, genetically or epigenetically modifying them in a controlled laboratory environment, and reintroducing them to the patient. For miRNA therapies, this process entails adjusting miRNA expression in these cells outside the body and confirming their therapeutic efficacy before transplantation [225]. 

This approach offers a high degree of precision and control over the cellular environment, allowing for careful monitoring and optimization of the miRNA expression. Researchers can ensure that only the desired miRNAs are active, and unwanted side effects can be detected before cells are reintroduced to the patient. Because cells are modified outside the body, they can be tailored to express specific miRNAs precisely, reducing the risk of off-target effects once transplanted. If the patient’s cells are used (autologous), the risk of immune rejection is minimized; however, using donor cells (allogeneic) can lead to immune reactions. In cancer therapies, such as chimeric antigen receptor T-cell (CAR-T) treatments, ex vivo miRNA manipulation can enhance the therapeutic efficacy of immune cells before reintroduction to the body [226]. 

Additionally, ex vivo techniques are beneficial for tissue regeneration applications where precise control over miRNA expression is required to promote the differentiation of stem cells into specific cell types, such as neurons, muscle cells, or hepatocytes. However, ex vivo manipulation is labor intensive and requires specialized cell cultures, gene editing, and reintroduction facilities. It involves multiple steps, including the extraction, manipulation, and transplantation of cells, which can be time consuming and expensive. Although this personalized approach can benefit individual patients, scaling up for widespread application presents significant challenges.

### 8.2. In Vivo Administration

In vivo administration directly delivers miRNA molecules, such as miRNA mimics or inhibitors, to a patient’s body using various delivery systems, including nanoparticles, viral vectors, or liposomes. This approach targets specific tissues or cells to modulate miRNA expression and achieve therapeutic effects. However, a significant concern with in vivo miRNA administration is the potential for miRNAs to inadvertently affect non-target genes in other tissues, leading to off-target effects. Despite advancements in delivery systems, such as tissue-specific nanoparticles, miRNAs may still reach unintended locations and alter the expressions of non-target genes. Additionally, direct administration of miRNA therapies can provoke immune responses, mainly when viral vectors are utilized, potentially causing inflammation or toxicity. Ensuring that miRNA therapy reaches only the intended cells or tissues remains challenging, as the body’s immune system, blood flow, and organ interactions complicate the precise control over miRNA delivery [227]. 

Nonetheless, in vivo miRNA therapies offer practical advantages, including less invasive administration methods, such as injections or oral delivery, making them more suitable for treating large populations. This approach requires fewer steps than ex vivo manipulation. It is generally more scalable, allowing for direct application to patients without cell extraction and re-infusion, expediting clinical treatments. In vivo administration is particularly suited for addressing systemic diseases where miRNA expression needs modulation throughout the body, such as metabolic disorders, like diabetes or cardiovascular conditions. Moreover, miRNA therapies targeting cancer or viral infections can be delivered in vivo to alter gene expression in tumors or infected tissues directly, and in vivo miRNA vaccines present a promising strategy for long-lasting immune protection against pathogens.

Although both ex vivo manipulation and in vivo administration have advantages, ex vivo manipulation offers a more controlled and precise approach, minimizing off-target effects and enhancing safety and specificity. In contrast, in vivo administration is more practical and scalable for treating widespread conditions; however, advancements in delivery technologies, such as tissue-specific nanoparticles, are essential to reduce off-target effects and improve the therapeutic precision [93].

The future of miRNA therapy will likely see a combination of these approaches, with improvements in delivery methods and gene-editing technologies helping to bridge the gap between the safety of ex vivo manipulation and the practicality of in vivo administration. Table 3 presents a comparison of ex vivo and in vivo developments.

### 8.3. Course of Action

The duration that an miRNA stays in tissue and whether it can be installed permanently in a specific tissue depend on several factors, including the delivery method, stability of the miRNA, tissue-specific degradation pathways, and whether the miRNA is introduced ex vivo or in vivo [228]. 

The stability of miRNA in tissues is influenced by its susceptibility to degradation by nucleases (enzymes that degrade RNA) and cellular recycling pathways. When miRNA mimics or inhibitors are delivered in vivo (directly to the body), their presence in the tissue is usually transient, often lasting from hours to days [229]. This duration depends on several key factors:

- Delivery System: If delivered via nanoparticles, liposomes, or viral vectors, the miRNA may be protected from immediate degradation, allowing for it to stay active longer. However, nucleases in the bloodstream or tissues rapidly degrade miRNAs delivered without protection;
- Tissue Type: Different tissues have varying miRNA turnover rates. For example, miRNAs in the liver quickly degrade because of high metabolic activity, while miRNAs in the brain or muscle may last longer because of different enzymatic environments;
- Endosomal Escape: For the miRNA to be active, it must escape the endosome after cellular uptake. If the delivery system is inefficient at facilitating endosomal escape, the miRNA may be degraded within the cell before it can act on its target mRNA;
- Half-life of MiRNA: Endogenous miRNAs typically have a half-life of 12–24 h, varying depending on the cellular environment. Synthetic miRNA mimics may be modified to enhance stability, but they still typically exhibit transient effects unless continuously delivered.

Overall, miRNAs delivered in vivo are generally temporary, requiring repeated administration for sustained effects. In contrast, ex vivo manipulation can offer a more permanent solution, especially when miRNAs are introduced as a part of a gene-editing or stable expression system as follows:

- Stable Expression Vectors: In ex vivo manipulation, miRNAs can be introduced using viral vectors (e.g., lentiviruses or AAVs) that integrate into the host cell’s genome, enabling continuous miRNA expression after the cells are transplanted back into the patient. In this case, the host cells would produce the miRNA indefinitely, offering a permanent or long-term solution;
- Lentiviral vectors, which integrate into the genome, can install miRNAs permanently in dividing cells, such as those in the liver or blood. However, this approach risks insertional mutagenesis (disruption of essential genes), making it less ideal for permanent in vivo miRNA therapy unless particular targeting strategies are employed;
- CRISPR-Based MiRNA Activation: Another ex vivo approach involves using CRISPR-based gene editing to permanently activate or repress specific miRNA genes within a cell’s genome. This would ensure the miRNA remains permanently active in the cells reintroduced to the patient. CRISPR/Cas9-based gene editing offers a theoretical path toward permanent miRNA installation in vivo. Using CRISPR/Cas9 to activate or repress the expressions of endogenous miRNAs directly, it may be possible to modulate miRNA expression within specific tissues permanently. For instance, CRISPRa (CRISPR activation) can upregulate the expressions of miRNAs that promote beneficial effects, such as those involved in muscle regeneration or cardioprotection. This approach would include delivering the CRISPR machinery and the guide RNA to specific tissues;
- Similarly, CRISPRi (CRISPR interference) can permanently repress miRNAs that cause harmful effects, such as miRNAs that promote fibrosis or inflammation. CRISPR can be delivered in vivo using lipid nanoparticles, plasmids, or other delivery vehicles that allow for the Cas9 protein and guide RNA (gRNA) to target specific cells, creating a permanent change in the genome. This approach provides a potential long-term solution for genetic diseases or tissue-specific modifications.

### 8.4. Synthetic mRNA Therapy with Self-Amplifying Systems

Synthetic mRNA therapy involves the delivery of messenger RNA (mRNA) that encodes for therapeutic proteins. Although traditional mRNA therapies are transient, recent advances have made it possible to extend the duration of mRNA expression. Self-amplifying RNA [230] is a next-generation mRNA technology that allows the delivered RNA to replicate within the cell, leading to prolonged protein expression without the need for viral vectors. SaRNA carries the genetic instructions for the desired therapeutic protein and the machinery to replicate itself in the target cell’s cytoplasm, allowing for extended therapeutic protein production. In cancer immunotherapy, saRNA can produce immune-stimulating proteins over an extended period, leading to long-term anti-tumor effects. SaRNA encoding muscle growth factors, like IGF-1, could sustain long-term muscle regeneration and hypertrophy [231].

### 8.5. Non-Viral Gene Therapy Systems for Long-Term Expression

Non-viral gene therapy methods, such as plasmid DNA or synthetic mRNA, can express miRNAs for extended periods but generally do not lead to permanent expression. However, new advances in synthetic RNA circuits that mimic natural transcriptional networks may allow for the long-lasting and tissue-specific control of miRNA expression, offering an alternative to viral vectors for long-term miRNA therapies. Despite the promise of these technologies, there are several challenges to achieving permanent installation of miRNAs in specific tissues ass follows [232]:

- Immune Response: One major challenge with permanent in vivo miRNA expression is the risk of triggering an immune response. Viral and non-viral delivery systems may activate the immune system, leading to vector clearance or potential damage to the host tissue. This is particularly relevant for viral vectors that persist in the body for long periods;
- Insertional Mutagenesis: If miRNA constructs are delivered using integrating viral vectors (such as lentiviruses), there is a risk of insertional mutagenesis, where the viral genome integrates into a critical region of the host’s genome, potentially disrupting essential genes and causing adverse effects, including cancer;
- Unintended Off-Target Effects: Permanently installing miRNA in a tissue could lead to off-target gene regulation, where the miRNA affects genes beyond the intended target. Although miRNAs are generally specific, they can target multiple genes, which raises the possibility of unintended consequences over the long term.

### 8.6. Nanoparticle-Based Delivery (Repeated Administration)

For repeated administration with more control, nanoparticles can deliver miRNA mimics or inhibitors directly to muscle tissues over time. These nanoparticles can be designed with ligands that bind specifically to muscle cells (such as the IGF-1 receptor), ensuring targeted delivery [202].

Nanoparticles can be engineered to slowly release miRNA mimics or inhibitors over time, providing sustained effects without frequent injections. This strategy would benefit athletes or individuals recovering from injury [233].

MRNA therapy is commonly delivered using lipid nanoparticles (LNPs), which protect the mRNA from degradation and facilitate cellular uptake. By designing LNPs with slow-release properties, it is possible to extend the duration of mRNA expression. In some cases, mRNA can be administered in a series of injections over time to achieve a sustained therapeutic effect.

Nanoparticle-based delivery systems can be designed to slowly release their therapeutic cargo over time, resulting in long-lasting effects. Polymeric nanoparticles can be engineered to encapsulate miRNAs, siRNAs, or other therapeutic molecules. These nanoparticles degrade slowly in the body, releasing their payload over weeks or even months. This approach is useful for achieving sustained therapeutic outcomes without repeated dosing. Polymeric nanoparticles could be used to deliver miRNAs that promote muscle regeneration, ensuring that the miRNAs are released gradually to support long-term muscle growth.

Hydrogels are another slow-release system that can be used to encapsulate therapeutic proteins, miRNAs, or growth factors. When implanted in or around the target tissue, hydrogels degrade slowly, releasing the therapeutic molecules over a prolonged period. A hydrogel with IGF-1 could be implanted in muscle tissue to promote long-term muscle regeneration following injury [234].

### 8.7. Cell-Based Therapies

Cell-based therapies involve transplanting genetically modified cells that can produce therapeutic proteins or miRNAs over an extended period. This approach is beneficial for achieving long-term effects in gene therapy or regenerative medicine [235].

Cells can be removed from the patient, genetically modified ex vivo (outside the body), and transplanted back into the patient. Once modified to express a therapeutic gene, these cells can produce the desired proteins or miRNAs over a long period. Muscle progenitor cells can be modified to overexpress muscle-specific miRNAs (e.g., miR-206) or growth factors (e.g., IGF-1), leading to long-term muscle repair and growth after reintroduction to the body. For diabetes, pancreatic cells could be modified to produce insulin or insulin-regulating miRNAs, providing long-term control over glucose levels. 

Induced pluripotent stem cells (iPSCs) can be genetically modified to express therapeutic genes or miRNAs. Once reintroduced to the patient, iPSCs can differentiate into the desired tissue type and continue to produce the therapeutic protein over a long period. IPSCs modified to express dopamine-producing enzymes could be used for the long-term treatment of Parkinson’s disease [236].

## 9. Discovery

Discovering new miRNAs involves a combination of high-throughput sequencing, bioinformatics analysis, and experimental validation. By applying these techniques, researchers can identify novel miRNAs in specific tissues or under particular conditions, contributing to a better understanding of their roles in gene regulation and disease. As sequencing technologies and computational tools evolve, the discovery of novel miRNAs will become even more efficient and widespread, allowing for more significant insights into miRNA biology across different organisms and tissues.

Integrated transcript annotation for small RNA (ITAS) represents a cutting-edge bioinformatics approach for accurately annotating small RNA (sRNA) transcripts, revolutionizing the understanding of their roles in gene regulation. Small RNAs, including microRNAs (miRNAs), small interfering RNAs (siRNAs), and Piwi-interacting RNAs (piRNAs), play critical regulatory roles in diverse biological processes by silencing target genes at the posttranscriptional level [237]. ITAS provides a comprehensive framework that integrates high-throughput sequencing data, such as small RNA-seq, with advanced algorithms to identify and annotate these RNA species with a high degree of precision [238]. Unlike traditional annotation tools, ITAS incorporates contextual information, such as sequence conservation, secondary structure prediction, and genomic features, ensuring the robust identification of sRNAs and their isoforms. For example, ITAS excels at differentiating mature miRNAs from their precursors and accurately mapping siRNA clusters, a task often challenging for conventional methods. This enhanced resolution is precious in uncovering novel sRNAs in complex organisms or understanding sRNA dynamics under specific conditions, such as stress or disease [239]. ITAS also integrates functional annotation, linking identified sRNAs to their putative targets and regulatory pathways, thus providing insights into their biological significance. The application of ITAS has already led to discoveries of novel miRNAs and siRNA families in plants and animals, highlighting its potential for broad biological and clinical research. By combining precision, scalability, and functional depth, ITAS transforms sRNA research, paving the way for discoveries in transcriptomics and RNA-based therapeutics.

High-throughput sequencing technologies, like RNA sequencing (RNA-Seq), are one of the most potent tools for miRNA discovery. NGS allows for sequencing all the small RNAs (18–25 nucleotides long) in a sample, including miRNAs, and provides a detailed landscape of the small-RNA population [240].

Key Steps:

- Isolate the total RNA from the tissue or cells of interest;
- Use specialized protocols to enrich small RNAs, including miRNAs (typically using size-selection methods);
- Prepare a small RNA library from the isolated RNA by ligating specific adapters to the 5′ and 3′ ends of small RNAs;
- Perform high-throughput sequencing on the small-RNA library. This generates millions of short reads corresponding to small RNAs present in the sample;
- Align the sequenced reads to the reference genome to identify known and potentially new miRNAs. Novel miRNAs can be identified by detecting sequences that match the criteria for miRNA precursor structures (such as the formation of a hairpin secondary structure);
- Once candidate miRNAs are identified through sequencing, northern blotting can be used to validate their expressions. This method detects small RNA molecules based on size and allows researchers to confirm the presence and abundance of a novel miRNA in a specific tissue or developmental stage;
- QPCR can be used to confirm the expression levels of newly discovered miRNAs.

Several computational tools and algorithms have been developed to predict novel miRNAs from sequencing data or genomic sequences. These tools typically use the following criteria for miRNA prediction:

- The presence of hairpin structures in precursor miRNAs (pre-miRNAs);
- The conservation of sequences across species;
- The minimum free energy (MFE) of the predicted secondary structure to determine if it will likely form a stable hairpin.

Standard miRNA Prediction Tools:

- miRDeep: A widely used algorithm that identifies novel miRNAs by aligning small-RNA reads to the genome and predicting precursor structures;
- miRBase: A comprehensive miRNA database that includes information on known miRNAs and can be used to cross-check potential novel miRNAs;
- RNAfold: A tool used to predict the secondary structure of RNA sequences, helping to determine if a candidate sequence forms a stable hairpin structure typical of miRNA precursors.

### 9.1. Comparative Genomics

Comparative genomics can identify novel miRNAs by analyzing the conservation of small RNA sequences across different species. MiRNAs conserved between species are more likely to have critical biological functions, and their identification can point to undiscovered miRNAs in less-studied species [241].

Steps:

- Align sequences from closely related species to find conserved regions of small RNAs;
- Analyze the conservation of the predicted secondary structures of the miRNA precursors.

### 9.2. Machine-Learning Models

Machine-learning techniques have become instrumental in discovering novel microRNAs (miRNAs). These models can predict new miRNAs across various genomes and tissues by analyzing features, such as nucleotide composition, sequence motifs, and the secondary structures of miRNA precursors [242]. 

Following the identification of a novel miRNA, functional validation is essential to elucidate its role and the target genes it regulates. Overexpression studies in cell cultures or animal models can reveal the miRNA’s impacts on gene expression and cellular phenotypes. Conversely, inhibition using antagomirs or small interfering RNAs (siRNAs) allows for assessing the effects of miRNA depletion [242]. 

MiRNAs typically regulate gene expression by binding to complementary sequences in the 3′ untranslated regions (3′ UTRs) of target messenger RNAs (mRNAs). Bioinformatics tools, like TargetScan and miRanda, predict potential mRNA targets based on seed sequence complementarity and evolutionary conservation [243]. 

Luciferase reporter assays are commonly employed to confirm that a novel miRNA regulates a predicted target gene. In this method, the 3′ UTR of the target mRNA is cloned downstream of a luciferase gene in a reporter vector; the binding of the miRNA to the 3′ UTR results in suppressed luciferase expression, leading to reduced luminescence. 

The discovery of miRNAs expressed at low levels or restricted to specific tissues or developmental stages presents challenges, often necessitating susceptible sequencing techniques or deep sequencing to detect these rare molecules [244]. Additionally, non-canonical miRNAs, which do not follow typical biogenesis pathways and may lack classic precursor hairpin structures, require specialized algorithms and experimental strategies for identification [245]. 

Given the complexity of the small-RNA transcriptome, sequencing can detect numerous small RNA fragments that are not genuine miRNAs. Therefore, applying stringent criteria during bioinformatics analysis and employing experimental validation methods, such as northern blotting or quantitative PCR (qPCR), are crucial to confirm the discovery of novel miRNAs [246].

## 10. Regulatory Process

As miRNA-based therapies advance, several ethical considerations must be addressed. A primary concern is the potential for off-target effects, which could lead to unintended gene silencing or overexpression. The precise manipulation of miRNA pathways to regulate gene expression raises questions about the long-term consequences of these interventions, particularly regarding heritable genetic changes. 

Although the potential of miRNA-based therapies and diagnostics is vast, challenges remain before these approaches become routine in clinical practice. A significant challenge is the need for comprehensive knowledge of miRNA off-target effects. MiRNAs often regulate multiple genes, and targeting a single miRNA could inadvertently affect unrelated biological pathways. Future research must improve the specificity of miRNA-based therapies to ensure they target only the desired pathways without causing unintended side effects [93]. 

Additionally, the regulatory landscape for miRNA-based therapies is still evolving. As these therapies become more sophisticated and widespread, regulatory agencies, like the FDA and EMA, must establish guidelines to ensure the safety and efficacy of miRNA-based treatments. This will likely involve stringent testing for off-target effects, toxicity, and long-term safety in clinical trials before miRNA therapies can be approved for widespread use. 

The future of miRNAs in medicine holds exciting possibilities. From their roles as non-invasive biomarkers to their potential in personalized therapeutics, miRNAs are poised to play transformative roles in precision medicine, cancer therapy, cardiovascular disease treatment, and regenerative medicine. As delivery technologies advance and our understanding of miRNA biology deepens, the ability to manipulate miRNAs safely and effectively will become increasingly feasible. 

Looking forward, miRNAs will likely become a cornerstone of personalized medicine, enabling more precise, targeted therapies tailored to individual patients’ genetic profiles. However, successfully integrating miRNAs into clinical practice will require continued innovation in delivery systems, safety mechanisms, and regulatory oversight. With these advancements, miRNA-based therapies have the potential to revolutionize the treatment of a wide array of diseases, offering hope for more effective and safer treatments in the future. 

## 11. Challenges and Limitations 

Despite the substantial progress in understanding microRNAs (miRNAs) and their therapeutic potential, several challenges and limitations persist. These challenges must be addressed before miRNA-based therapies can be widely adopted in clinical practice. The complexities of miRNA biology, the issues related to delivery mechanisms, and the risks of off-target effects and immune responses are just a few of the hurdles researchers face. This section explores the primary challenges that limit the application of miRNA therapies and diagnostics while also considering the innovative approaches being developed to overcome these obstacles.

One of the most significant challenges in miRNA research is the inherent complexity of miRNA regulation. MiRNAs can target multiple genes, and a single miRNA may regulate hundreds of different mRNAs. This characteristic makes miRNAs highly versatile but challenges remain in predicting their exact roles in biological processes. The interactions between miRNAs and their targets are often context dependent, meaning the same miRNA can have different effects in different cell types or under various physiological conditions [247].

This complexity creates difficulties in designing targeted miRNA therapies, as it is often challenging to predict how modulating one miRNA will affect other pathways. Moreover, the regulation of miRNAs is influenced by the tissue-specific expression of their target mRNAs, the presence of competing endogenous RNAs (ceRNAs), and RNA-binding proteins that can modulate miRNA activity. These factors complicate the development of therapeutics because they require a detailed understanding of the miRNA’s role in the specific disease context [248].

Additionally, although bioinformatics tools have improved our ability to predict miRNA targets, these predictions are unreliable. Experimental validation of miRNA-mRNA interactions is often required, adding complexity and time to developing miRNA-based therapies [249].

### 11.1. Off-Target Effects and Immune Responses

MiRNAs often regulate multiple genes, and this multi-targeted nature raises concerns about off-target effects. When administering miRNA mimics or inhibitors, there is a risk that they could unintentionally alter the expression of genes unrelated to the target disease pathway, leading to unintended consequences. Off-target effects are particularly concerning in cancer therapy, where the goal is to specifically target tumor cells without affecting healthy cells. Unintended gene silencing in normal cells could disrupt essential biological processes and lead to toxicity [250].

Additionally, synthetic miRNA therapeutics, such as miRNA mimics or antagomirs, can stimulate immune responses. Some synthetic RNA molecules are recognized as foreign by the immune system, triggering an immune reaction that can lead to inflammation and other adverse effects. This is particularly relevant when using viral vectors, which are highly immunogenic. Efforts to minimize these immune responses include developing chemically modified miRNAs, such as those with 2′-O-methyl or locked nucleic acids [189], which are less likely to activate the immune system.

### 11.2. MiRNA Stability and Degradation

The instability of miRNAs in the body is another limitation that complicates their therapeutic use. In the bloodstream, nucleases rapidly degrade miRNAs, making it difficult to maintain therapeutic concentrations at the target site. MiRNA mimics and antagomirs must be protected from degradation by chemical modification or encapsulation in protective carriers, such as nanoparticles or liposomes. However, these strategies complicate drug development and increase production costs [251].

Stability issues also affect the use of circulating miRNAs as biomarkers. Although miRNAs are more stable than mRNAs, they are still subject to degradation, which can complicate their detection and quantification in body fluids, like blood, saliva, or urine. This has implications for the reliability of miRNA-based diagnostic tests, especially in early-stage disease detection.

## 12. Intellectual Property

New miRNA discoveries, including their sequences, functions, and therapeutic applications, can be patented if they meet the criteria for patentability (novelty, non-obviousness, and utility). Although naturally occurring miRNAs may face challenges in being patented in some jurisdictions, innovative applications, modifications, and delivery systems for miRNAs remain patentable. Researchers and companies must carefully navigate existing patents and prior art to ensure their miRNA-related inventions are protectable and commercially feasible.

New findings related to miRNAs, including their sequences, functions, and potential therapeutic applications, can be patented, provided they meet the necessary criteria for patentability. However, like any other intellectual property, there are specific legal and regulatory considerations regarding patenting miRNAs, and some miRNA-related inventions may already be patented.

For a new miRNA discovery or related application to be patented, it must meet the following general criteria, similar to those for patenting any biological innovation: novelty, non-obviousness, and utility. Several aspects of miRNA discoveries can be patented. Novel miRNA sequences can be patented, especially those not previously identified or functionally characterized. This applies to both the mature miRNA sequence and the precursor miRNA sequence (pre-miRNA). However, patents for naturally occurring sequences may face challenges, particularly in some jurisdictions, where laws may restrict the patenting of naturally occurring biological molecules without significant modification.

Patents for miRNA-based therapeutic applications, such as miRNA mimics or inhibitors (antagomirs) used to treat diseases, can be granted. Also, delivery systems for miRNA therapeutics, such as nanoparticles or viral vectors, and methods for using miRNAs to regulate gene expression in specific tissues for therapeutic purposes, such as promoting muscle growth or inhibiting tumor growth, can be patented.

Patents can cover using miRNAs as biomarkers for diagnosing diseases, monitoring disease progression, or predicting patients’ responses to treatments. For example, miRNA expression profiles could be patented for non-invasive cancer screening or diagnosing neurodegenerative disorders.

Identifying novel target genes regulated by miRNAs, particularly if the target interactions were previously unknown and significantly impacted gene regulation or diseases, can also be patented. This is particularly relevant if the target gene is linked to a specific therapeutic strategy or diagnostic approach.

Innovative delivery systems designed specifically for miRNA therapies, including modified nanoparticles, lipid carriers, or tissue-targeted vectors, can be patented if they demonstrate novel and non-obvious delivery capabilities.

Modifications of natural miRNAs to improve their stability, specificity, or therapeutic effect—such as chemically modified nucleotides, engineered stem–loop structures, or synthetic miRNAs—can be patented, as they are considered as non-naturally occurring and involve human intervention.

Biotech companies and research institutions have already patented many miRNAs, their functions, and therapeutic applications. For example, companies, like miRagen Therapeutics, Regulus Therapeutics, and Alnylam Pharmaceuticals, have patented various miRNA therapeutics, diagnostic uses, and delivery methods. Regulus Therapeutics has filed patents on the use of specific miRNAs as biomarkers for diseases, even though the miRNA sequences are naturally occurring. These patents cover specific applications in diagnosing diseases, like hepatitis C or cardiovascular conditions, based on miRNA expression profiles.

In some jurisdictions, such as the United States, laws and precedents can limit the patenting of naturally occurring biological sequences. For example, in the landmark Association for Molecular Pathology v. Myriad Genetics, Inc. (2013) case, the U.S. Supreme Court ruled that naturally occurring DNA sequences cannot be patented, although modified or synthetic sequences may still be eligible. Similar considerations may apply to naturally occurring miRNA sequences, meaning patent protection may be difficult if the miRNA is unmodified.

Even if an miRNA is novel, it may be difficult to patent if related sequences, uses, or applications have been described in prior art. Before the patent application is filed, the prior art includes any publicly available information related to the miRNA, its sequence, or potential uses. Patent offices carefully review prior art to ensure the invention is genuinely new.

### Examples of miRNA Patents

Patent applications have been filed for miRNA mimics and inhibitors targeting various diseases, such as cancer (e.g., targeting miR-21 or miR-155) or cardiovascular conditions (e.g., targeting miR-208a for heart failure).

MiRNA Biomarkers: Regulus Therapeutics has filed patents on using specific miRNAs as biomarkers for diseases, even though the miRNA sequences are naturally occurring. These patents cover specific applications in diagnosing diseases, like hepatitis C or cardiovascular conditions, based on miRNA expression profiles. Patents have been granted for using miRNAs as biomarkers for diseases, like liver cancer (e.g., miR-122 as a diagnostic marker), or assessing heart damage post myocardial infarction (e.g., miR-1 and miR-133).

MiRNA Delivery Systems: Patents exist for delivery systems explicitly designed for miRNA-based therapies, such as lipid nanoparticles or biodegradable polymers, for targeted delivery to specific tissues (e.g., muscle or liver).

Combinations of miRNAs or miRNAs with other therapeutic agents can also be patented. These combinations can create new, non-obvious therapeutic strategies that leverage the regulatory roles of multiple miRNAs in different biological pathways. For example, a patent could claim a combination of miR-1 (for cardiac repair) and miR-206 (for muscle regeneration) in a therapeutic approach to treat multiple organ systems after a traumatic injury. The delivery system used to deliver miRNA-based therapies can be patented even if the miRNA itself is naturally occurring. Delivery systems often play a critical role in the success of miRNA therapies, especially in ensuring tissue-specific targeting, enhanced stability, and controlled release. Patents on delivery systems could include nanoparticles engineered to deliver miRNAs specifically to the heart, brain, or muscles; viral vectors; or other carriers designed to enhance the uptake and efficacy of miRNA-based therapies in target tissues.

Companies, like Dicerna Pharmaceuticals, have developed and patented specialized lipid nanoparticle delivery systems for miRNA therapeutics. These nanoparticles improve the stability and bioavailability of the miRNA in vivo, which is patentable even if the miRNA sequence itself is not.

Another way to patent miRNAs is by developing synthetic miRNA analogs similar to naturally occurring miRNAs but engineered with differences in sequence, structure, or function. These synthetic miRNAs may have enhanced properties, such as greater specificity for target genes, better stability, or improved efficacy.

MiRNA sponges or decoys are synthetic constructs that bind to and inhibit specific miRNAs, thus preventing them from acting on their target mRNAs. These constructs can be patented if designed in a novel and non-obvious way.

The patentability of miRNAs, mainly naturally occurring ones, can vary significantly by jurisdiction. Although natural miRNA sequences face challenges in the U.S. and the European Union, there are some distinctions in patent laws globally as follows:

United States: Following the Myriad decision, naturally occurring biological sequences are generally not patentable unless modified or applied in a novel and non-obvious way. However, therapeutic applications and delivery systems remain patentable.

European Union: Like in the U.S., the Biotechnology Directive prohibits patents on naturally occurring biological sequences unless an industrial application is demonstrated. However, synthetic or modified miRNAs, delivery methods, and therapeutic uses are patentable.

India: Patent laws in India are stricter regarding biological substances. Section 3(j) of the Indian Patent Act excludes “plants and animals in whole or in any part thereof other than microorganisms but including seeds, varieties, and species, and essentially biological processes for producing or propagating plants and animals” from patentability. However, modified or synthetic miRNAs and novel applications of miRNAs may still be patentable.

China: China’s patent laws allow for the patenting of modified biological sequences and applications of miRNAs in diagnostics and therapeutics, making it more permissive in some respects than other jurisdictions.

## 13. Commercial Products

Several companies are actively developing miRNA-based products (Table 4).

## 14. Conclusions

We foresee that many other areas of RNA research deserve attention, which may lead to more Nobel Prizes in this field and, indeed, expand the utility of RNA therapies.

- miRNA Rejuvenation of Therapeutic Protein Expression: In therapeutic protein production, controlling the consistent and long-term expressions of proteins, especially in cases like gene therapies, is essential for achieving a sustained therapeutic effect. miRNAs could be used to rejuvenate or regulate the expressions of proteins significantly when their production naturally declines because of aging or disease [252];
- Enhancing Protein Expression in Aging Tissues: Aging often leads to a decline in the production of key proteins, which can contribute to diseases, such as sarcopenia, neurodegeneration, and metabolic disorders. MiRNA therapy could be designed to rejuvenate these therapeutic proteins’ expressions by suppressing protein production inhibitors or activating the necessary transcription factors. For example, the downregulation of miR-34a, which is associated with aging, could be employed to increase the expression of sirtuins (proteins involved in longevity and metabolism) or factors that promote muscle regeneration, like IGF-1 (insulin-like growth factor) [253];
- Controlling Protein Production in Gene Therapy: Ensuring that the therapeutic gene is expressed at the right time and in the right amount is crucial. MiRNA-based systems could precisely control protein expression by designing synthetic miRNA circuits that respond to environmental or cellular signals. For instance, miRNA switches could be used to control the expression of therapeutic proteins only when needed, reducing side effects and improving the overall safety profile of gene therapies. This could be particularly beneficial in conditions requiring intermittent treatment, such as intermittent hormone deficiencies or enzyme replacement therapies [254];
- miRNAs in Enhancing Protein Stability and Folding: miRNAs can be used to regulate chaperone proteins involved in folding therapeutic proteins. In cystic fibrosis, the correct folding of proteins is critical, and mutations can lead to misfolding. MiRNA-based therapies could target pathways that enhance the production of molecular chaperones, improving the folding and function of therapeutic proteins [255];
- MiRNA-Based Vaccines: Vaccines are traditionally designed to elicit immune responses against specific pathogens by presenting the body with an antigen (such as a weakened virus or protein subunits) [256]. However, miRNA technology could open new frontiers in vaccine development, offering several advantages, including enhanced specificity, long-lasting immune responses, and the potential to create personalized vaccines. One of the most exciting applications of miRNA technology in vaccines involves creating vaccines that induce host cells to produce antigens from within. By designing a vaccine that introduces miRNAs targeting viral RNA or mRNA encoding viral proteins, host cells can produce specific viral antigens, stimulating the immune system more naturally. This approach could benefit rapidly mutating viruses, such as influenza or SARS-CoV-2. A synthetic miRNA-based vaccine could target conserved regions of the viral genome, minimizing the effects of viral mutations. This would provide longer-lasting immunity and eliminate the need for frequent updates to vaccine formulations. Cancer vaccines stimulate the immune system to recognize and attack tumor-specific antigens. MiRNA technology could enable the creation of personalized cancer vaccines by targeting tumor-specific mutations.
- Tumors often downregulate or mutate proteins that suppress tumor growth, such as p53, or proteins that would usually alert the immune system to cancerous cells. MiRNA-based vaccines could enhance the expression of these proteins in tumor cells, increasing the likelihood that the immune system will detect and destroy them [257]. Another futuristic application is using miRNAs to boost the presentation of antigens in traditional vaccines. Antigen-presenting cells (APCs) play a crucial role in the immune response by displaying antigens to T-cells. MiRNAs could enhance these cells’ function by upregulating key pathways involved in antigen processing and presentation. For instance, miR-155 is involved in immune cell activation, and its upregulation could enhance the effectiveness of vaccine-induced immune responses. By co-delivering miRNAs that boost antigen presentation with traditional vaccines, it may be possible to create vaccines that provide more robust, longer-lasting immunity [258];
- miRNA in Plant-Based Vaccines: MiRNAs could also play an essential role in producing plant-based vaccines, which use plants to produce antigens that can be administered orally or through traditional injection. Researchers could enhance the yield and stability of antigen production in plants by manipulating miRNAs in plant systems. For example, miRNAs that regulate protein synthesis or stress responses in plants could be targeted to increase the efficiency of vaccine antigen production in edible crops, such as lettuce or tomatoes. This approach could pave the way for inexpensive and easily scalable vaccines suitable for global use, particularly in resource-limited settings [259].

MicroRNAs (miRNAs) have emerged as versatile tools in stem cell biology and regenerative medicine, offering potential therapeutic strategies for various diseases and tissue repair scenarios. Their applications reflect the cutting-edge potential of RNA research, with future discoveries poised to impact medicine, therapeutics, and molecular biology significantly. However, the successful implementation of miRNA-based therapies faces several challenges, including the complexity of miRNA biology, difficulties in targeted delivery, risks of off-target effects and immune responses, and concerns about stability and degradation. Addressing these issues is crucial for miRNA-based therapies’ safe and effective transition from research to clinical practice. 

The future of miRNA research in medicine is promising. MiRNAs serve as non-invasive biomarkers for early disease detection and offer personalized therapeutic interventions, potentially improving human health. As delivery technologies and molecular design strategies advance, miRNA-based therapies will likely become integral in treating various diseases, including cancer, cardiovascular disease, neurodegenerative disorders, and viral infections. Integrating miRNA data into precision medicine, alongside developments in synthetic biology and regenerative medicine, will further expand the range of miRNA applications. Nonetheless, overcoming challenges related to specificity, delivery, and ethical considerations remains essential to ensure the safety and efficacy of miRNA-based therapies. 

MiRNAs are rapidly emerging as powerful tools against various diseases, with several miRNA-based therapeutics already in clinical trials. They offer novel approaches to targeting the underlying genetic mechanisms of diseases, such as cancer, cardiovascular diseases, viral infections, and fibrotic disorders. However, challenges related to delivery, immune responses, and off-target effects must be addressed before miRNA therapies can become standard clinical practice. As research progresses and more advanced delivery systems are developed, the potential for miRNA-based therapies to revolutionize personalized medicine and improve patient outcomes becomes increasingly apparent. 

The future of miRNA-based therapies is promising, with personalized medicine expected to play a significant role in their development. As miRNA-profiling technologies improve, clinicians can identify specific miRNAs involved in a patient’s disease and design tailored therapies targeting those miRNAs. This approach could revolutionize the treatment of complex diseases, like cancer, where individual tumors often exhibit unique miRNA expression patterns. Additionally, combination therapies that pair miRNA therapeutics with other treatments, such as small-molecule inhibitors or immunotherapies, are expected to become more prevalent. By targeting multiple pathways simultaneously, these combination approaches could enhance treatment efficacy and reduce the likelihood of drug resistance, particularly in diseases like cancer and viral infections [171]. 

Despite their promise, the clinical development of miRNA-based therapies faces several challenges. One primary hurdle is delivering miRNA mimics and antagomirs to target tissues while avoiding off-target effects. Systemic delivery can spread throughout the body, increasing the risk of off-target gene silencing and potential toxicity. Researchers focus on developing more targeted delivery systems, such as lipid nanoparticles (LNPs) and exosome-based carriers, to deliver miRNAs directly to affected tissues. Another challenge is managing immune responses triggered by synthetic miRNA molecules. For instance, the immune-related adverse events observed in clinical trials of MRX34 were a significant setback [93]. 

The development of miRNA-based therapies also faces regulatory challenges. As miRNAs are relatively new to the therapeutic landscape, regulatory agencies, like the FDA and EMA, still establish guidelines for their approval and use. Ensuring that miRNA-based treatments meet safety and efficacy standards requires rigorous preclinical and clinical testing, which can be time consuming and costly. Ethically, concerns exist about the long-term effects of manipulating gene expression through miRNA modulation. Altering miRNA levels could have unintended consequences for off-target genes, leading to unforeseen health issues, mainly when used in germline editing or hereditary disease interventions. As miRNA therapies move closer to clinical implementation, addressing these ethical concerns through appropriate regulatory frameworks and informed consent processes is crucial [105,120,260].

## Figures and Tables

**Table 1:** RNA types, prevalence, and recognitions.

Type of RNA	Function	Percentage of RNA	Nobel Prize Details
RNA Discovery	Enzyme polynucleotide phosphorylase is responsible for RNA synthesis.	--	1959: For discovering the mechanisms of RNA and DNA synthesis
Messenger RNA (mRNA)	Serves as the template for protein synthesis during translation	1–5%	1961 Nobel Prize in Physiology or Medicine to François Jacob and Jacques Monod for discovering mRNA’s role in protein synthesis
Transfer RNA (tRNA)	Carries amino acids to the ribosome during translation	10–15%	1968 Nobel Prize in Physiology or Medicine to Robert W. Holley, Har Gobind Khorana, and Marshall W. Nirenberg for their interpretation of the genetic code and its function in protein synthesis, including tRNA discovery
RNA as a Catalyst	Catalysis function of RNA	n/a	1989: Sidney Altman and Thomas R. Cech for discovering that RNA can act as a catalyst, leading to the understanding of ribozymes
Small Nuclear RNA (snRNA)	Involved in the splicing of pre-mRNA by forming the spliceosome complex	<0.1%	1993 Nobel Prize in Physiology or Medicine to Richard J. Roberts and Phillip A. Sharp for discovering split genes and RNA splicing involving snRNA
Small Interfering RNA (siRNA)	Involved in RNA interference, degrading complementary mRNA to regulate gene expression; primarily involved in defense against viruses	<0.1%	2006 Nobel Prize in Physiology or Medicine to Andrew Fire and Craig Mello for discovering RNA interference (RNAi), of which siRNA is a significant component
Ribosomal RNA (rRNA)	Forms the structural and catalytic components of ribosomes, essential for protein synthesis	80–90%	2009 Nobel Prize in Chemistry to Venkatraman Ramakrishnan, Thomas A. Steitz, and Ada E. Yonath for studies of the structure and function of the ribosome
CRISPR-Cas9 using RNA	Gene-editing technology using RNA to guide DNA modification	n/a	2020: Jennifer A. Doudna and Emmanuelle Charpentier for developing CRISPR-Cas9, a gene-editing technology
MicroRNA [1]	It regulates gene expression by binding to target mRNAs and promoting their degradation or inhibiting translation.	<0.1%	2024 Nobel Prize in Physiology or Medicine to Victor Ambros and Gary Ruvkun for the discovery of microRNA and its role in posttranscriptional gene regulation, revealing a new layer of gene regulation that impacts development and disease
Long Noncoding RNA (lncRNA)	Regulates gene expression at various levels, including chromatin remodeling, transcription, and posttranscriptional processing	<1%	No Nobel Prize directly for lncRNA, though it is a rapidly evolving field.
Circular RNA (circRNA)	Acts as an miRNA sponge and may regulate gene expression; it is stable because of its circular structure.	<1%	No Nobel Prize is related to circRNA yet, but research is ongoing and rapidly evolving.
Piwi-Interacting RNA (piRNA)	Protects the germline by silencing transposable elements; mainly found in reproductive cells	Variable, from <1% to higher than 1%	No Nobel Prize specific to piRNA, but RNA-interference-related discoveries were recognized by the 2006 Nobel Prize.
Small Nucleolar RNA (snoRNA)	Guides chemical modifications of other RNAs, particularly rRNA	<0.1%	No Nobel Prize was specific to snoRNA, but it was linked to rRNA function, and it was recognized by the 2009 Nobel Prize in Chemistry for ribosome research.
Antisense RNA (asRNA)	Inhibits gene expression by binding to complementary mRNA, preventing translation or inducing degradation	<0.1%	There is no Nobel Prize specifically for antisense RNA, but the field of RNA-based gene silencing has greatly expanded based on earlier work on RNA interference.

**Table 2:** Main databases of miRNA.

Database Name	Hyperlink	Description
miRBase	https://www.mirbase.org/ (accessed on 12 October 2024)	A primary miRNA sequence database that provides annotations, references, and information about miRNA families
miRTarBase	https://mirtarbase.cuhk.edu.cn/ (accessed on 12 October 2024)	Contains information on experimentally validated miRNA–target interactions compiled from the literature
TargetScan	https://www.targetscan.org/ (accessed on 12 October 2024)	Provides predicted miRNA targets based on conserved binding sites and includes information about miRNA conservation
miRDB	http://mirdb.org/ (accessed on 12 October 2024)	A database for predicted miRNA targets using a machine learning approach, with options to query miRNA functions
DIANA-miRPath	http://diana.imis.athena-innovation.gr/DianaTools/index.php (accessed on 12 October 2024)	Provides pathway-based miRNA functional analysis by linking miRNA target genes to known pathways
HMDD (Human MicroRNA Disease Database)	https://www.cuilab.cn/hmdd (accessed on 12 October 2024)	A manually curated database that collects miRNA–disease associations from the scientific literature
miRGator	http://mirgator.kobic.re.kr/ (accessed on 12 October 2024)	An miRNA analysis tool that integrates expression data, functional annotation, and predicted targets
miREnvironment	https://www.tools4mirs.org/ (accessed on 12 October 2024)	Focuses on miRNA–environment interactions, allowing users to explore how miRNAs are affected by environmental factors
mir2Disease	http://www.mir2disease.org/ (accessed on 12 October 2024)	A curated database that links miRNAs to various human diseases based on experimental evidence
OncomiRDB	http://lifeome.net/database/oncomirdb/ (accessed on 12 October 2024)	A specialized database that focuses on miRNAs implicated in cancer biology and therapeutics
miRCancer	http://mircancer.ecu.edu/ (accessed on 12 October 2024)	Focuses on miRNAs and their involvement in various types of cancer, with data on expression and regulation
miRGeneDB	https://mirgenedb.org/ (accessed on 12 October 2024)	A database providing curated annotations of miRNA genes across different species, focusing on high-quality annotations

**Table 3:** Comparison of ex vivo manipulation and in vivo administration.

Criteria	Ex Vivo Manipulation	In Vivo Administration
Precision and Control	High degree of precision and control because of manipulation in a lab setting	Less control: delivery systems face challenges in targeting specific tissues.
Safety and Off-Target Effects	There is a lower risk of off-target effects; cells are screened before reintroduction.	Higher risk of off-target effects and immune responses due to systemic delivery
Efficiency and Practicality	It is time consuming, expensive, and more challenging to scale for large populations.	Faster and more accessible to administer; more practical for large-scale applications
Applications	Best for cell-based therapies, regenerative medicine, and personalized treatments	Suitable for systemic diseases, cancer therapy, and infectious diseases

**Table 4:** MiRNA-based products.

Regulus Therapeutics
RG-012: An miRNA inhibitor targeting miR-21, developed for treating Alport syndrome, a genetic kidney disease. It is currently in Phase II clinical trials.
RG-125 (AZD4076): Developed in collaboration with AstraZeneca, targeting miR-103/107 to treat non-alcoholic fatty liver disease (NAFLD)
miRagen Therapeutics
Cobomarsen (MRG-106): An anti-miR-155 therapy targeting cutaneous T-cell lymphoma (CTCL); it has reached Phase II clinical trials.
Remlarsen (MRG-201): An miRNA mimic of miR-29, designed to prevent fibrotic diseases, such as pulmonary and skin fibrosis
Alnylam Pharmaceuticals
Onpattro (Patisiran): An FDA-approved siRNA-based transthyretin-mediated amyloidosis (ATTR) therapy
ALN-PCSsc: An experimental therapy targeting PCSK9 for hypercholesterolemia.
Dicerna Pharmaceuticals
DCR-PHXC is an RNAi therapeutic targeting HAO1 for primary hyperoxaluria in Phase III trials.
DCR-HBVS: An RNAi therapeutic for hepatitis B (HBV) in Phase I/II trials
Santaris Pharma (now a part of Roche)
Santaris developed miravirsen (SPC3649), an anti-miR-122 therapy for Hepatitis C. It was among the first miRNA-based therapies to enter clinical trials, reaching Phase II.
Rosetta Genomics
Diagnostic tools using miRNA biomarkers; its miRview^®^ assays are used for the diagnosis of cancers, like lung cancer and mesothelioma.
MiRXES
GASTROClear, an miRNA biomarker assay for the early detection of gastric cancer. This diagnostic tool has been commercialized in Asia and is considered as a leading non-invasive test for gastric cancer screening.
Gene Signal
Aganirsen is an anti-miR-21 therapy aimed at treating ocular neovascularization, a process involved in diseases like age-related macular degeneration.
Hummingbird Bioscience
HMBD-001: This product targets HER3-driven cancers, utilizing RNA interference (RNAi) technology, which modulates gene expression through siRNA or miRNA. This is currently in the preclinical stage.
Storm Therapeutics
STC-15: An RNA-based therapy targeting specific miRNAs involved in cancer progression. This product is still in the preclinical development stage.
Marina Biotech
CEQ508: This is an miR-34 mimic designed to treat familial adenomatous polyposis (FAP), a condition that increases the risk for developing colorectal cancer. It is currently in Phase I clinical trials.
Silence Therapeutics
SLN360: This product is designed to target lipoprotein(a), a known risk factor for cardiovascular diseases, using siRNA-based gene-silencing technology. It is currently in Phase I clinical trials.
Viridian Therapeutics
VRDN-001: This siRNA-based therapy is in the preclinical phase and aims to treat autoimmune diseases, such as thyroid eye disease, by modulating RNA pathways.

## Data Availability

Not applicable.
